# The hidden fluorine: a critical review of analytical methods for unmasking PFAS and closing fluorine mass balance in environmental samples

**DOI:** 10.1007/s00216-026-06458-6

**Published:** 2026-04-05

**Authors:** Damalka Balasuriya, John Michael N. Aguilar, Alexander C. Hoepker, Joshua S. Wallace, Diana S. Aga

**Affiliations:** 1 Department of Chemistry, The State University of New York – University at Buffalo, Buffalo, NY 14260 USA; 2 Research and Education in eNergy, Environment, and Water (RENEW) Institute, The State University of New York - University at Buffalo, Buffalo, NY 14260 USA

**Keywords:** Total fluorine, Organofluorine quantitation and speciation, EOF, AOF, TOF

## Abstract

Per- and polyfluoroalkyl substances (PFAS) are recognized as persistent environmental contaminants, yet their regulatory definitions differ substantially. The U.S. Environmental Protection Agency (EPA) applies a narrow structural definition, whereas the Organization for Economic Co-operation and Development (OECD) adopts a broader framework encompassing millions of fluorinated species. This discrepancy directly affects which compounds are monitored and quantified, leaving many environmentally relevant fluorochemicals, such as trifluoroacetic acid (TFA), refrigerants, and other organofluorine (OF) compounds, unaccounted for. Compared to the OECD’s classification of PFAS, which can include up to 7 million species, the EPA definition only covers about 14,000 PFAS. Recent studies reveal that currently known PFAS explain only a small fraction of the total organofluorine (TOF) in environmental samples, underscoring the limitations of liquid chromatography with tandem mass spectrometry (LC-MS/MS) commonly used for targeted analysis. This review provides critical insights on other analytical methods that are underutilized for PFAS analysis, including combustion ion chromatography (CIC), fluorine-19 nuclear magnetic resonance spectroscopy (^19^F-NMR), particle-induced gamma-ray emission (PIGE), and x-ray photoelectron spectroscopy (XPS), that can directly or indirectly measure fluorine. By highlighting their advantages and limitations, we propose how these tools can complement mass spectrometry to close the fluorine mass balance and improve monitoring of PFAS and related fluorochemicals. Finally, while solid-phase extraction (SPE) is a useful tool for matrix cleanup and analyte concentration, it is not appropriate for total fluorine determination because many compounds may be lost throughout the process. Alternatively, direct analysis and other extraction techniques are presented for better fluorine mass balance coverage.

**Graphical Abstract:**

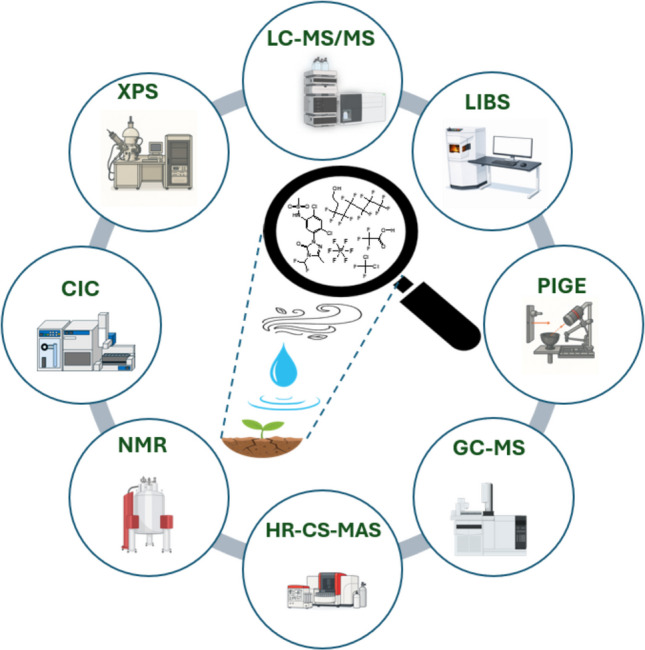

## Introduction

Per- and polyfluoroalkyl substances (PFAS) have emerged as a global environmental concern due to their persistence, high mobility, bioaccumulative properties, and documented toxicity [1]. Therefore, the use of PFAS has increasingly been restricted globally, and the manufacture of certain classes of PFAS has been phased out in many countries. Effective control of restricted PFAS in products requires enforcement systems supported by reliable analytical methods capable of measuring these substances across diverse matrices. A comprehensive report from the Nordic Council of Ministers highlights current analytical limitations and outlines the regulatory conditions needed to strengthen compliance verification under existing and future PFAS restrictions [2]. Since then, formal definitions of PFAS have continued to evolve across agencies to guide monitoring and risk assessment efforts. Notably, the United States (US) Environmental Protection Agency (EPA), under the Toxic Substances Control Act, defines PFAS based on the presence of at least one of the following structures: (1) R–(CF_2_)–CF(R′)R″, where both the –CF_2_– and –CF– moieties are saturated carbons; (2) R–CF_2_OCF_2_–R′, where R and R′ can be F, O, or saturated carbons; and (3) CF_3_–C(CF_3_)R′R″, where R′ and R″ can be F or saturated carbons [3]. In contrast, the Organization for Economic Co-operation and Development (OECD) offers a broader definition, describing PFAS as fluorinated substances that contain at least one fully fluorinated methyl (–CF_3_) or methylene carbon (–CF_2_–) not attached to H, Cl, Br, or I atoms [4]. This divergence in definitions underscores ongoing debates about the scope of PFAS classification and the implications for environmental monitoring and regulatory policy.

**Fig. 1:**
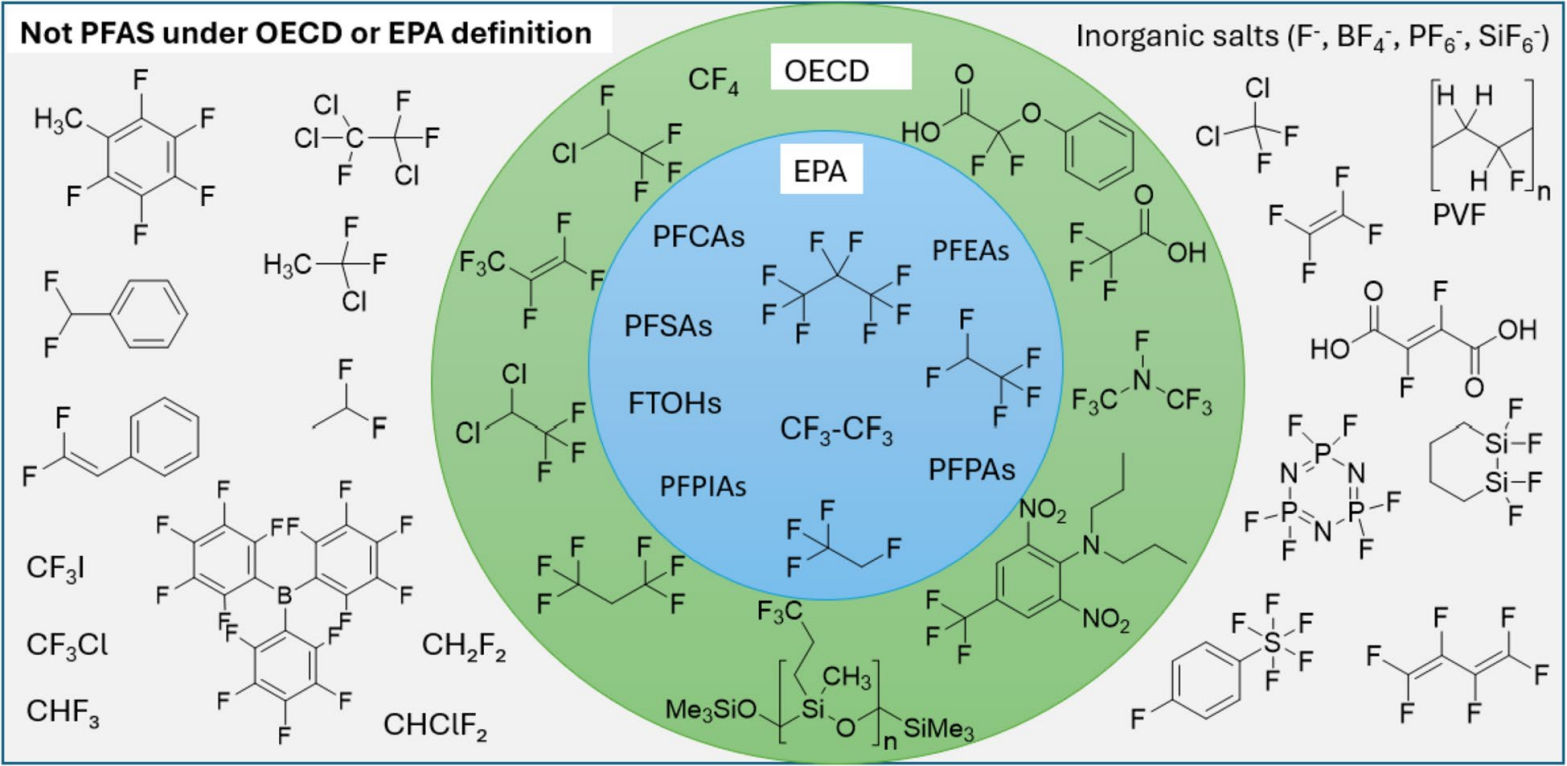
Venn diagram of compounds falling under EPA and OECD definitions, demonstrating overlap, disparities, and chemistries requiring additional analysis. Perfluoroalkyl carboxylic acids (PFCAs), perfluoroalkyl sulfonic acids (PFSAs), perfluoroether acids (PFEAs), perfluoroalkyl phosphonic acids (PFPAs), perfluoroalkyl phosphinic acids (PFPIAs), fluorotelomer alcohols (FTOH)

Because these definitions serve as regulatory tools, the narrow scope of the EPA’s working definition, which is far more restrictive than the OECD’s classification, has been widely criticized by the scientific community [5]. The EPA’s definition focuses on specific structural motifs, excluding thousands of known fluorinated chemicals, many of which are environmentally persistent and toxic. This structural narrowness creates a regulatory blind spot, allowing many “forever chemicals” to evade oversight. Notably, the EPA definition omits many fluorochemicals (Fig. 1) that have been well-documented as environmental contaminants, such as trifluoroacetic acid (TFA) and refrigerants like perfluoromethane (CF_4_), 2,2-dichloro-1,1,1-trifluoroethane (R-123), 2-chloro-1,1,1,2-tetrafluoroethane (R-124), and tetrafluoropropene. While some of these are considered industrial gases or non-traditional PFAS, they share key environmental characteristics with regulated PFAS,including extreme persistence, potential for long-range transport, and, in some cases, degradation into toxic perfluorinated acids [6]. Compared to the OECD’s classification of PFAS that can include as many as 7 million species [4, 5], the EPA definition only includes about 14,000 PFAS [7].

**Fig. 2:**
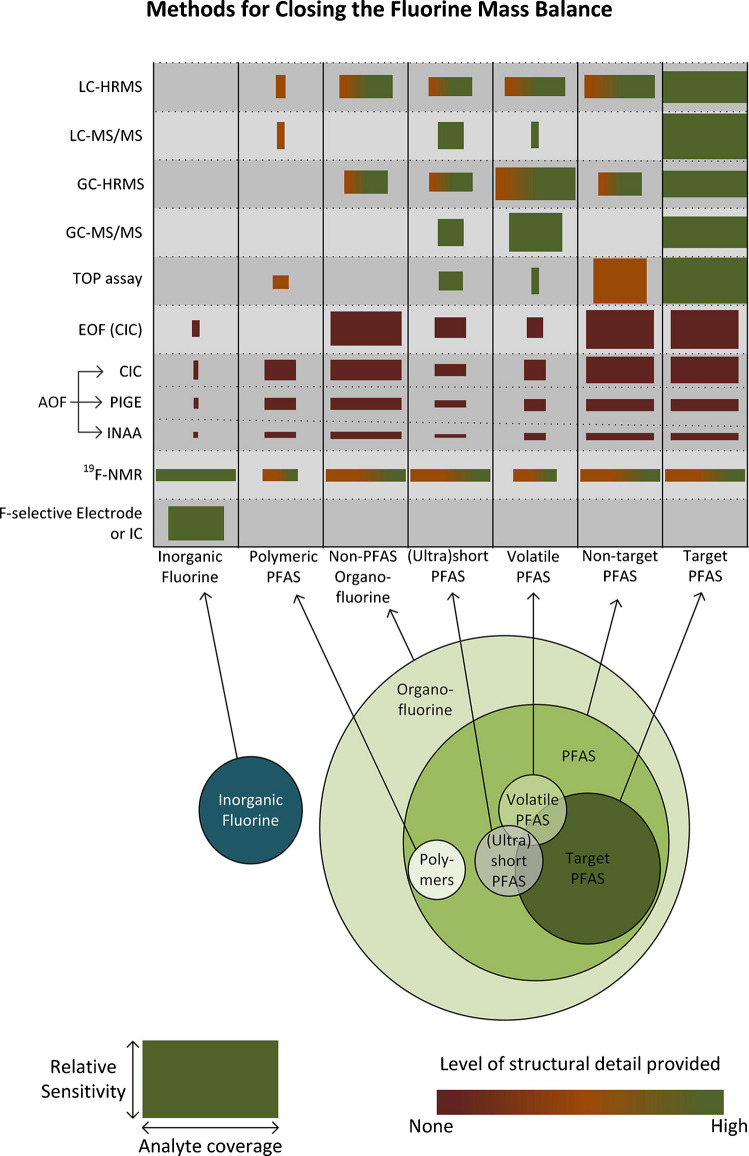
Overview of the relative potential of analytical methods to complete the fluorine mass balance in PFAS destruction, for different types of fluorinated substances potentially found in environmental samples. “Target PFAS” are the typically < 100 specific PFAS included in a GC- or LC-MS analyte list, for which standards are available. “Polymeric PFAS” include fluoropolymers, perfluoropolyethers, and side-chain fluorinated polymers and may be present in environmental samples as micro- or nanoparticles. The heights of the boxes represent the general sensitivity of the method as typically employed, and the widths the general coverage of the method within the corresponding group of fluorinated substances. These parameters also strongly depend on the sample types (air, water, soil, etc.), sample collection (grab vs integrated and whole air vs sorbents), and preparation methods (extraction and concentration), which are not included in the figure and can vary significantly depending on the practitioner. Ideally, EOF and AOF methods detect only organic fluorine, but because the extent of fluoride removal is often slightly below 100%, these methods are shown to include some inorganic fluorine. Reprinted from: Smith, S.J., et al. “The Need to Include a Fluorine Mass Balance in the Development of Effective Technologies for PFAS Destruction.” *Environmental Science & Technology* 58.6 (2024): 2587–2590. Licensed under CC BY [18]

While targeted analysis using liquid chromatography (LC) coupled with tandem mass spectrometry (MS/MS) remains the “gold standard” for quantifying known PFAS and is essential for regulatory testing, toxicology screening, and human health evaluations, the technique captures only a narrow subset of PFAS. For instance, the US EPA method 1633A targets only 40 PFAS, and is used for monitoring those with established drinking-water maximum contaminant levels: 4 ppt for perfluorooctanoic acid** (**PFOA) and perfluorooctane sulfonate (PFOS) and 10 ppt for perfluorononanoic acid, perfluorohexanesulfonic acid, and hexafluoropropylene oxide dimer acid (commonly known as GenX) [8]. However, many analytical tools capable of detecting a broader range of PFAS and other organofluorine (OF) compounds are underutilized. Because regulatory and analytical definitions determine which compounds are targeted for detection, they inherently constrain our understanding of the occurrence, diversity, and environmental behavior of persistent and potentially toxic fluorinated species. Therefore, this review aims to critically evaluate the advantages and limitations of complementary analytical approaches that can enhance the detection, characterization, and quantification of PFAS and other OF compounds, thereby contributing to a more complete fluorine mass balance in environmental and biological samples (Fig. 2).

To account for a wide range of “PFAS” this review paper will adopt the newer, broadly inclusive PFAS definition by the OECD [4] when discussing appropriate methodologies, which includes any fluorinated substances that contain at least one fully fluorinated methyl or methylene carbon, with the few noted exceptions [4]. A more comprehensive inclusion is justified by the cumulative contribution to the overall fluorine burden and the potential for some OF compounds that are non-PFAS to degrade into traditional PFAS or other potentially harmful transformation products in biological systems, the environment, or wastewater treatment plants [9, 10].

Even when targeted LC–MS/MS analysis is complemented by non-targeted LC–high-resolution mass spectrometry (LC-HRMS), a substantial portion of OF compounds in many samples remains uncharacterized. This gap arises because non-targeted workflows still depend on existing suspect lists and spectral libraries, leaving numerous PFAS outside these lists and non-PFAS OF compounds undetected. For example, Aro et al. [11] found that 84% of the EOF in human blood was unidentified, likely reflecting the presence of PFAS not included in targeted methods and their transformation products. By definition, OF compounds isolated through extraction procedures such as solid-phase extraction (SPE) are classified as EOF [12]. Although non-targeted LC–HRMS has enabled the discovery of many previously unrecognized PFAS, the resulting measurements are often semi-quantitative due to the lack of authentic reference standards, preventing closure of the fluorine mass balance. Recent studies underscore the magnitude of this challenge: Ruyle et al. [10] reported that regulated PFAS accounted for less than 10% of the EOF in influent and effluent from US municipal wastewater treatment plants. Similarly, De Vega et al. [13] employed a multi-platform analytical approach for ski wax products and found that targeted PFAS quantified by LC–ion mobility spectrometry (IMS)–HRMS accounted for less than 1% of the EOF. Complementary methods, including pyrolysis GC–MS, GC with atomic emission detection (AED), combustion ion chromatography (CIC), inductively coupled plasma mass spectrometry, and fluorine-19 nuclear magnetic resonance (^19^F-NMR), were necessary to characterize the broader suite of fluorinated compounds. Together, these findings highlight the essential role of complementary analytical techniques in achieving a more complete understanding of PFAS occurrence and the overall fluorine mass balance.

In this review, we will discuss direct (extraction-free) analytical techniques that are less commonly used but are highly valuable for measuring OF in samples, including CIC, ^19^F-NMR, particle-induced gamma-ray emission spectroscopy (PIGE), and X-ray photoelectron spectroscopy (XPS) (Table 1).  Additional techniques, including high-resolution continuum-source molecular absorption spectroscopy (HR-CS-MAS), and laser-induced breakdown spectroscopy (LIBS)will also be discussed later. We will compare these methods with widely used indirect (extraction-based) methods that require sample preparation for quantification, such as LC-MS/MS, GC-MS, and the total oxidizable precursor (TOP) assay. This review does not include advances in targeted and non-targeted analysis using LC or GC coupled to MS because several recent reviews focusing on these topics have already been published (Baqer et al. [14], Koch et al. [15], Shen et al. [16], Teymooriyan et al. [17]). We will provide examples of successful applications of the less utilized techniques for analyzing fluorine-containing compounds and suggest how they can be employed effectively as complementary tools to close the fluorine mass balance in specific studies where fluorine accounting is critical, such as assessing efficiencies of PFAS remediation or in monitoring studies that detect high amounts of EOF that cannot be explained by targeted LC-MS/MS analysis.

**Table 1:** Direct total fluorine measurement methods by analytical strategy

Method	IF vs OF speciation	Sample type	Destructive	Notes	Instrumental limits of detection (LOD)
CIC	✗	Solids/liquids	Yes	Enables EOF and AOF analysis with matrix limitations	5.5–21 ppb^a^ (TF) [19]
XPS	✓	Solids (cryo-probe required for liquids)	No	Direct chemical speciation; surface and bulk characterization; high LOD for liquids	0.05–0.1 atom % (500–1000 ppm [25]) 20 ng on substrate (SPE) [26]
PIGE	✗	Solids/filters liquids	No	Surface-specific, non-destructive	36–239 ppm (0.13–0.86 µg/cm^2^)^b^ [15] 50 ppt (SPE) [27]
^19^F-NMR	✓	Solids/liquids	No	Excellent speciation; quantitative	10–100 ppb [28–31] in solution

^a^Detection limits for CIC are generally reported in units of ng F. For comparison, reported values were converted to ppb (ng/g) assuming a typical aqueous volume of 0.20 mL and a density of 1.0 g/mL. CIC inherently quantifies total fluorine released per sample, independent of absorbate volume, dilution, or sample heterogeneity with an LOD of 1.1–4.2 ng F.

^b^Detection limits for PIGE are generally reported in units of µg F/cm^2^. For comparison, reported values were converted to ppm (µg/g) here and below assuming typical operational parameters for PIGE analysis, including a sampling depth of 30 µm, irradiated area of 2 mm, and sample density of 1.2 g/cm^3^ for a low-density organic environmental matrix. Actual values may vary due to differences in sampling depth and sample density.

## Total fluorine analysis in environmental samples

**Fig. 3:**
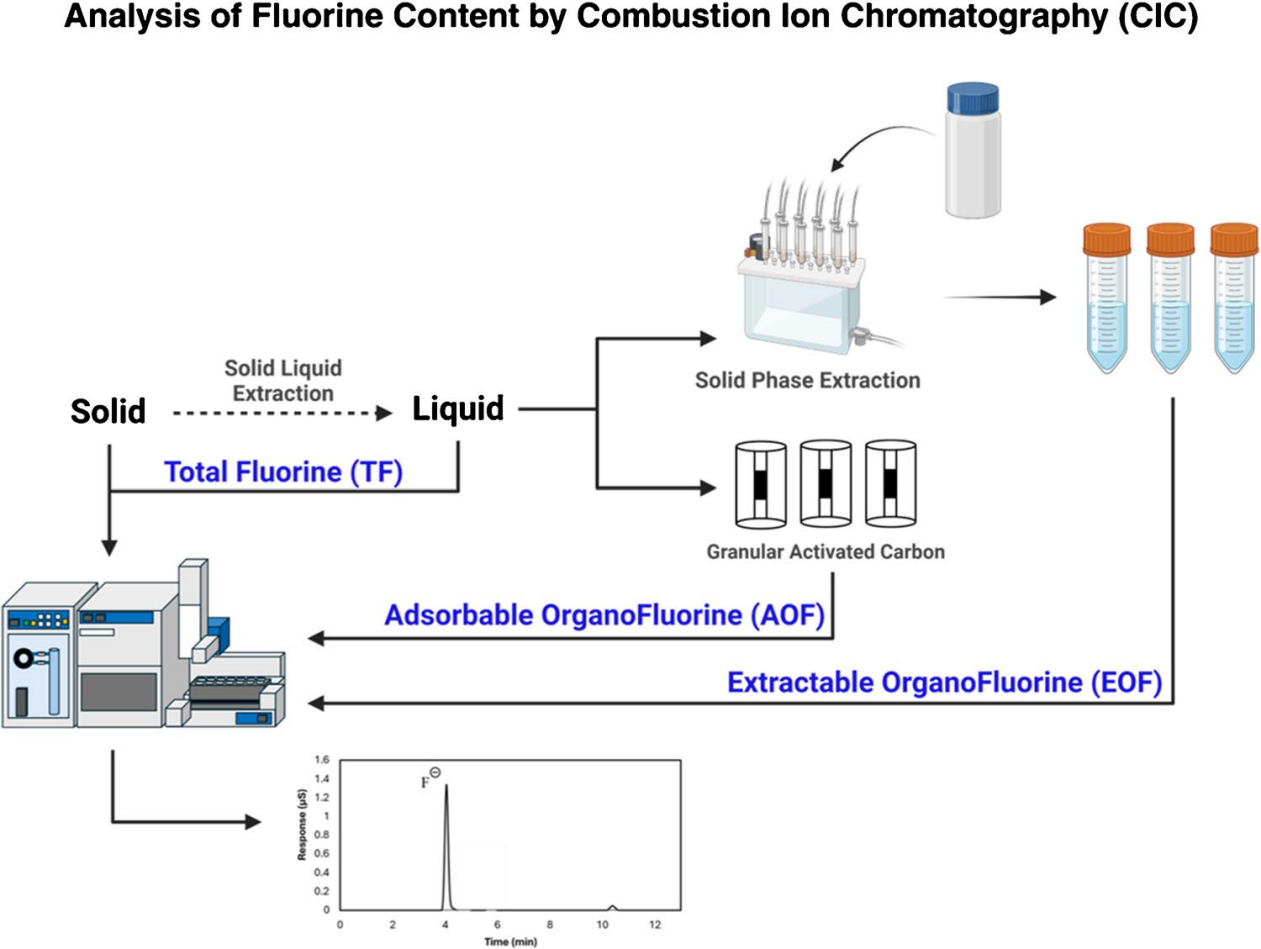
Overview of fluorine content analysis using combustion ion chromatography (CIC). Direct CIC analysis provides the total fluorine (TF) content, which includes both inorganic fluorine (IF) and total organofluorine (TOF). TOF can be further divided into: (1) adsorbable organofluorine (AOF), the fraction of organofluorine (OF) that can be retained from liquid samples on activated carbon [23], and (2) extractable organofluorine (EOF), the portion of OF that can be isolated from samples through extraction techniques such as solid–liquid, liquid–liquid, or solid-phase extraction [19]

The accurate quantification of total fluorine (TF) in environmental samples, both liquid and solid, can be achieved using direct analysis by CIC, as shown in Fig. 3. Methods for direct TF analysis with minimal sample treatment should be prioritized to prevent analyte loss. Minimal treatment methods, such as filtration, centrifugation, or temperature-controlled lyophilization, are useful because they preserve a broader range of fluorinated species, including small ions, volatile components, polymers, and other poorly soluble compounds that might otherwise be lost during extraction. However, direct TF analysis alone is not sufficient for a complete evaluation of fluorine content because natural and engineered samples may contain substantial levels of inorganic fluoride (IF), which can dominate TF signals and interfere with OF analysis. Therefore, the distinction between OF and IF is crucial for characterizing total (T)OF compounds and ultimately for regulating PFAS. Indirect fluorine analysis, performed after sample extraction or adsorption into granular activated carbon (GAC), provides information on the amount of TOF. When analyzed by CIC, the eluate from SPE provides the EOF content, while combustion of adsorbed components in GAC provides adsorbable (A)OF content [19] (as shown in Fig. 3). Understanding the TOF composition is essential for tracing contaminants back to their source, enabling the development of effective remediation plans and helping pinpoint the parties responsible for contamination. However, characterizing EOF, which is commonly done using targeted LC-MS/MS and nontargeted LC-HRMS analysis [20], often results in a large discrepancy between the fluorine levels obtained by CIC and those measured by the latter methods.

Some of the compounds may be lost during SPE, leading to an underestimation of EOF because they have poor retention in the sorbent materials, which depends heavily on the type of sorbent used [21]. While the use of GAC to measure AOF captures ionic, zwitterionic, and neutral species, highly polar compounds such as TFA do not adsorb effectively, resulting in an underestimation of the OF content in the sample [22]. The limitations of indirect fluorine analysis have been demonstrated in previous studies in which both EOF and AOF have been reported to underestimate TOF [14], mostly due to the losses of ultrashort- and short-chain PFAS [21]. 

The difference between TF and TOF content in samples is usually attributed to the presence of IF and other highly polar OF compounds that are not retained in GAC or SPE [24]. Despite their importance, there are no standard methods for determining TF or TOF in environmental samples [18].

## Overview of techniques used for measuring TF and TOF

The use of CIC for measuring TF in solid, semi-solid, and liquid samples involves combustion at high temperatures (900–1050 °C) in an oxygen-rich environment, converting all fluorinated compounds to hydrogen fluoride (HF), which is absorbed in water, converted to fluoride ions at pH > 6, and quantified by IC [19]. This approach quantifies TF by capturing all fluorine-containing compounds—including those undetectable by LC-MS/MS—under conditions where combustion efficiency for C_4_–C_12_ PFAS is generally consistent and minimally affected by co-occurring compounds [31]. However, CIC cannot distinguish OF from IF or identify individual OF species [11], and it can lose volatile compounds prior to combustion, causing substantial underestimation of TF when volatile OF fractions are abundant [19].

For liquid samples, AOF can be determined after the sample is passed through GAC, which has a high surface area, porosity, and pH-dependent surface charge that enable strong PFAS adsorption, as described in EPA Method 1621 [19]. GAC surfaces are generally negatively charged under neutral to basic conditions and positively charged in acidic conditions. However, since common PFAS (e.g., perfluoroalkyl carboxylic acids (PFCAs) and perfluoroalkane sulfonic acids (PFSAs)) have very low pK_a_ values and remain deprotonated across typical environmental pH ranges [32], acidification of samples to pH 2 to increase electrostatic interactions with GAC does not change the ionic charge of most PFAS.

Improving AOF measurements requires redefining their analytical scope to better capture the soluble TOF fraction, including ultrashort-chain, volatile, cationic, and other emerging OF species. Priority needs include standardizing GAC properties, establishing validated breakthrough criteria (especially for ultrashort-chain PFAS), and testing alternative sorbents (e.g., mixed-mode or ion-exchange) to improve retention of hydrophilic and volatile compounds. Using AOF alongside EOF can strengthen fluorine mass-balance assessments. Recent refinements, includinglarger sample volumes (up to 500 mL) and tandem GAC columns, have boosted adsorption capacity, improved recoveries, and increased agreement between EOF and targeted PFAS across diverse matrices [33].

**Fig. 4:**
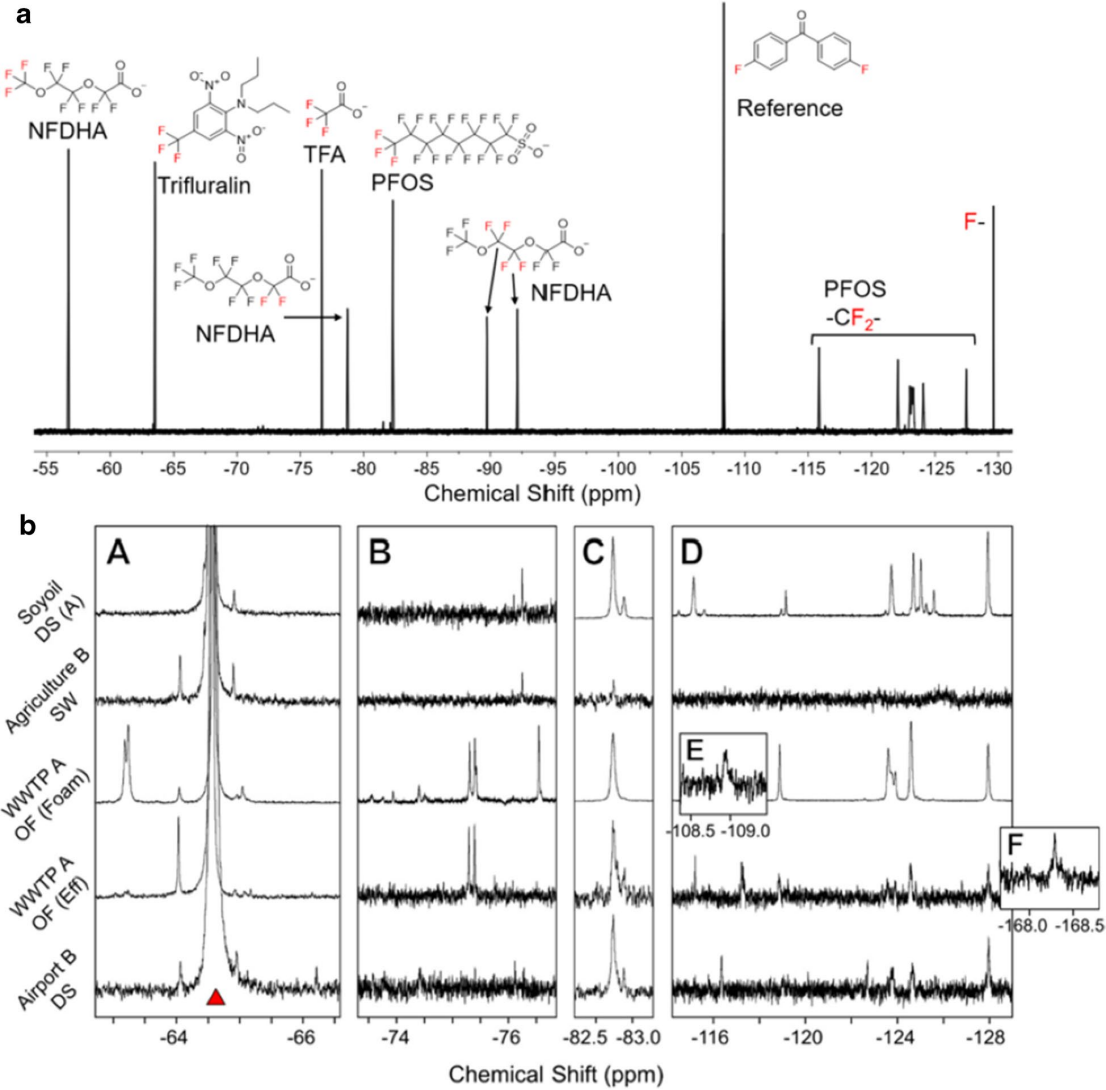
**a** Conceptual ^19^F-NMR spectrum of selected fluorinated compounds. The spectrum shows characteristic chemical shifts for PFAS and related compounds. Peaks are assigned to N-Fluoro-2,2-difluoro-2-hydroxyacetamide (NFDHA, −57 to −93 ppm, multiple signals), Trifluralin (~ −67 ppm), TFA (~ −76 ppm), PFOS (−82 ppm, −115 ppm, and −125 ppm), and inorganic fluoride (F^−^; ~ −129 ppm). A reference standard (2,2′-difluorobenzophenone) is shown at −108 ppm. **b** Representative ^19^F-NMR spectra in portions of characteristic chemical shift regions: (i) Aryl CF_3_, − 70 to − 60 ppm; (ii) CF_3_, − 80 to − 70 ppm; (iii) PFAS CF_3_, − 85 to − 80 ppm; (iv) CF_2_, − 130 to − 110 ppm. Samples: surface water downstream from the Soyoil facility (Soyoil DS (A)), surface water in an agricultural area (Agriculture B SW), foam at wastewater treatment plant (WWTP) outfall (WWTP A OF (Foam)), effluent at a WWTP outfall (WWTP A OF (Eff)), and surface water downstream from an airport (Airport B DS). Insets show minor resonances observed for the (v) WWTP A OF (Foam) sample at − 108.9 ppm (below signal-to-noise cutoff of 3) and (vi) WWTP A OF (Eff) sample at − 168.3 ppm. The large peak indicated with a red triangle at − 64.6 ppm is the SPE recovery spike of 3,5-Bis(trifluoromethyl)benzoic acid (BTFMBA). Reprinted with permission from: Faber, Katharine A., et al. [31] “Revealing Organofluorine Contamination in Effluents and Surface Waters with Complementary Analytical Approaches: Fluorine-19 Nuclear Magnetic Resonance Spectroscopy (^19^F-NMR) and Liquid Chromatography-Tandem Mass Spectrometry (LC-MS/MS).” *Environmental Science & Technology *59.28 (2025): 14695–14706. Copyright 2026 American Chemical Society

^19^F-NMR spectroscopy is a non-destructive technique that utilizes a strong magnetic field to align fluorine nuclei, which are then perturbed by radio-frequency excitation. The resulting signal is produced by relaxing precession of the net magnetization vector back to equilibrium, which induces a detectable voltage in the receiver coil at specific chemical shifts dictated by the local electronic environment of fluorine nuclei. When used for TF analysis, it is a top-down method detecting all fluorine, requiring retrospective signal assignment to identify compounds. This untargeted capability is one of ^19^F-NMR’s core strengths, especially for discovery-based research. The wide spectral width (~ + 100 to −300 ppm) generally provides excellent peak resolution; however, the discrete separation of possibly hundreds of individual OF compounds is not feasible. Significant spectral overlap is expected in complex mixtures, particularly within the internal -CF_2_- regions (typically between −115 and −130 ppm). Crucially, the utility of ^19^F-NMR in this context is not centered on the complete characterization of every fluorinated molecule, but rather on the quantification of fluorine chemical classes. For example, the terminal -CF_3_ group in PFAS exhibits a characteristic resonance at ~ −75 to −85 ppm (Fig. 4a). Integration of these resonances relative to an internal reference standard allows the measurement of the molar concentration of total CF_3_-bearing PFAS without requiring molecular identification [28, 34, 35]. As demonstrated by Faber et al. [31], this class-based fluorine speciation enables direct quantification of major fluorinated groups based on their characteristic chemical shift signatures (Fig. 4b). Distinct ^19^F resonances were used to quantify aryl-CF_3_ groups (−70 to −60 ppm), PFAS terminal –CF_3_ groups (−85 to −80 ppm), PFAS –CF_2_ units (−130 to −110 ppm), and aliphatic –CF_3_ groups, including TFA and perfluorinated alcohols (−80 to −70 ppm). This class-based quantification strategy provides a powerful means to assess the distribution of fluorine across different chemical classes without requiring identification of individual compounds [31]. Knowledge of the fluorine count per fluorine resonance allows the molar TF estimation of each fluorine class.

^19^F-NMR readily detects the fluorine resonances of ultrashort-chain PFAS [35], and high molecular weight fluoropolymers [36] that are often missed by LC-MS/MS and the TOP assay (see TOP assay section below). Camdzic et al. compared ^19^F-NMR with the results of the TOP assay and found that ^19^F-NMR consistently reported higher TOF concentrations—63% more than the TOP assay followed by LC-MS/MS analysis, and 65% more than targeted analysis and suspect screening using LC-HRMS [35]. Similarly, ^19^F-NMR analysis of water samples revealed substantially higher TF than the PFAS quantified by targeted LC-MS/MS, which accounted for only 3.7–27% of the ^19^F-NMR-determined TF [37]. In a recent study demonstrating near-complete mineralization of PFAS by sonolysis, ^19^F-NMR played a crucial role in providing fluorine mass balance by simultaneously detecting both the OF, including the terminal -CF_3_ peak typical in most PFAS, and the IF produced during degradation [38].

Generally, ^19^F-NMR is about 1000-fold less sensitive than MS-based techniques, and therefore it has not been widely used for environmental analysis. A limit of detection (LOD) of 10 ppb for PFOS can be achieved in 45 min using 4 mg/mL Cr(acac)_3_ as an NMR relaxation agent to increase acquisitions per time [28]. To improve LODs, 16-h acquisitions can be performed to achieve LODs of around 1–2 ppb. To further address the sensitivity limitation, ^19^F steady-state free precession (SSFP) NMR was developed in combination with complete reduction to amplitude frequency tables (CRAFT) analysis that enables hundreds of acquisitions in the time of a single conventional scan, thereby enhancing ^19^F-NMR sensitivity per unit time [29]. For example, spiked PFOA in human serum showed ~ 50 × lower LOD compared to conventional ^19^F-NMR. Furthermore, sub-ppb LODs for TFA has been achieved (< 1 ppb in 15 min), and LODs as low as 80 ppt for TFA have been reported in extended (24-h) scans using SSFP ^19^F-NMR [30]. With modern ≥ 800 MHz cryogenically cooled NMR systems, further sensitivity improvements of 5–10 × over 500 MHz instruments are expected [30].

For solid samples, the direct analysis of fluorine content can be achieved using solid-state (SS) ^19^F-NMR. Thijs et al. [39] detected PFOA spiked onto paper (17 ppm total fluorine), achieving a 5-10 ppm LOD with a 20-h acquisition. This study resolved –CF_2_– and –CF_3_ signals at a S/N ratio of ~ 25 using a 4 mm rotor at 15 kHz Magic Angle Spinning on a 500 MHz Bruker Avance III spectrometer. Typical sample requirements range from 5 to 100 mg, depending on the rotor size. To date, this remains the only published application of SS-^19^F-NMR for detecting and quantifying fluorinated compounds in solid matrices. While the inherent sensitivity of SS NMR is lower than that of liquid-state NMR (~ 10^2^–10^3^-fold), the development of Dynamic Nuclear Polarization (DNP) offers a significant opportunity to overcome this limitation. DNP can enhance signal sensitivity by factors typically exceeding 100-fold, and in some cases up to 200-fold [31, 40]. To our knowledge, DNP remains an unexploited opportunity for the direct analysis of fluorinated compounds in the solid state.

**Fig. 5:**
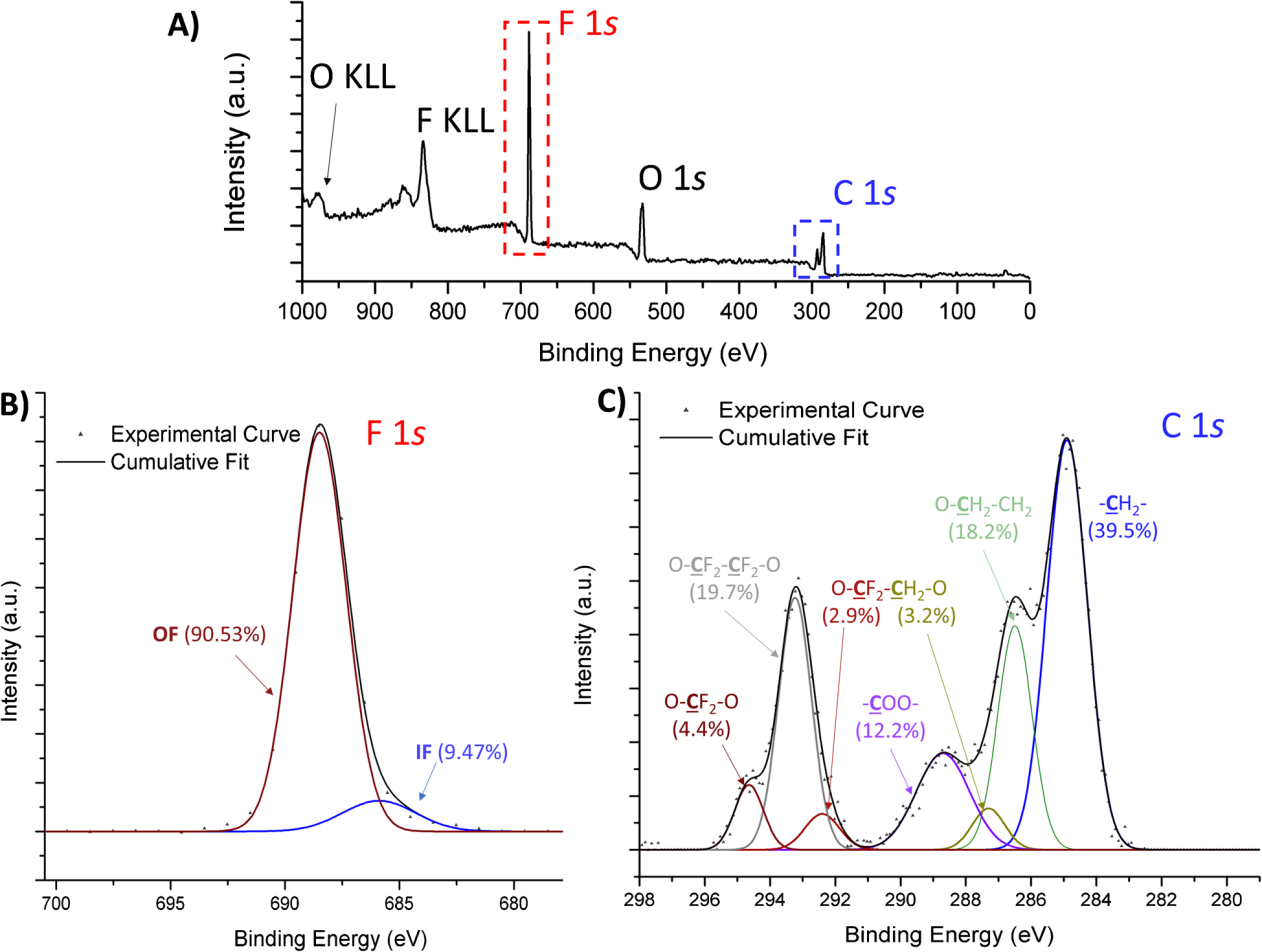
XPS analysis of a mixed fluorinated polyether (PFPE) (1% w/w) on the surface of poly(ε-caprolactone) demonstrating: **A** the presence of fluorine (F 1*s*, red), oxygen (O 1*s*), and carbon (C 1*s*, blue) in a survey scan; **B** the quantitative differentiation of organic (90.53% of F) and inorganic fluorine (9.47%) in a high-resolution F 1*s *scan of the final polymer; and **C** quantitative disambiguation of seven C- and C-F functional groups in a high-resolution C 1*s *scan. (Original data)

XPS, a surface-sensitive technique used to determine the elemental composition, chemical states, and electronic structure of materials within the top 5–10 nm of a sample’s surface, can also be used for TF analysis. Direct surface analysis provides valuable insight into surface contamination, coatings, and fluorine-related surface reactions. Despite recent advances in solution-phase and combustible analyses, critical analytical limitations remain for non-(N)-EOF compounds, insoluble inorganics, and non-combustible analytes. Direct quantitative analysis and speciation of OF and IF are achieved by probing C 1*s* and F 1*s* electron binding energies. During XPS analysis, samples are irradiated with soft X-rays under vacuum, and the generated photoelectrons provide element- and environment-specific kinetic energies, enabling both qualitative and quantitative assessment of fluorine and C-F moieties on sample surfaces (Fig. 5) [41, 42].

There are several advantages to XPS as a complementary analytical tool, including direct, non-destructive analysis of TF and OF, and simultaneous reporting of chemical state and quantity information for all co-occurring chemistries, such as the sulfonates in per- and polyfluorosulfonates (S 2*p*), and extensive publicly available chemical shift libraries [43]. The advantages provided by direct analysis of insoluble or non-combustible materials make XPS an essential complement to other TF analytical tools. Tokranov et al. [42] utilized XPS to quantify fluorine in various consumer products, revealing that textiles treated with PFAS contained up to 45 atom % fluorine at the surface, demonstrating that XPS can detect fluorine concentrations as low as 16 nmol/cm^2^. Similarly, Wu et al. [44] used XPS to screen for fluorotelomer-based polymers in child car seats that could not be analyzed by traditional extraction methods. Modern instruments further allow for chemical state mapping [45] and can provide important insights into OF sorption and binding [46], degradation mechanisms [47], and fluorine segregation and enrichment [41]. Additionally, soluble analytes in environmental samples, including polymeric OF, can be concentrated and cast onto a substrate for analysis [41]. However, special care is required for samples, extracts, and analytes not intrinsically compatible with ultra-high vacuum environments. Commercially available cryo-probes achieving temperatures below −100 °C enable direct analysis of samples containing more volatile OF compounds. Bulk analysis can also be achieved through depth profiling or cross-sectional analysis wherein the total quantity of fluorine species can be evaluated as a function of depth from the surface and volume of material removed during sputtering.

PIGE spectroscopy has also recently been adapted to quantify TF in solid samples, including N-EOF [48]. In PIGE, an accelerated proton beam excites nuclei in a targeted sample—whether solid surfaces, pelletized powder samples or adsorbed liquid samples on appropriate sorbents—causing element-specific gamma emissions (for fluorine at 110 and 197 keV) whose intensities are proportional to the fluorine content [15, 27]. Similar to XPS, PIGE is gaining popularity for TF analysis because it is non-destructive, requires limited sample preparation, exhibits matrix-independent ppm sensitivities (68–238 ppm or 13–45 nmol F/cm^2^) and can provide complementary information about the co-occurring elements [48, 49]. However, PIGE has not yet been widely adopted because it cannot distinguish between OF and IF, requires highly specialized equipment to generate the incident ion beam [15], and is sensitive to sample heterogeneity. PIGE has been applied as a rapid screening method for PFAS in water using gravity filtration through an activated carbon felt, enabling TF detections at levels below 0.05 ppb in 2 L of water [27].

The utility of PIGE as a complement to traditional analyses was demonstrated by Wu et al. [44], which demonstrated large differences between TF determined by PIGE (497 ppm) and LC-MS/MS (31 ppm) in fluorotelomer-based polymers prior to extraction for targeted analysis, likely due to the limited solubility and ionizability of fluoropolymers during MS analysis [44]. In 2020, a field-deployable PIGE system was developed to screen for PFAS in groundwater at aqueous film-forming foam (AFFF)-impacted sites using a portable proton accelerator, achieving low-ppb (1.1–1.4 ppb) detection limits for EOF during analysis of the sorbent packing following SPE [48, 49]. PIGE analysis of TF directly in paper and textile samples similarly exhibited LODs of 68 ppm (13 nmol F/cm^2^) and 127–238 ppm (24–45 nmol F/cm^2^), respectively, minimizing bias from extraction and enabling efficient screening across consumer products [50]. Rodowa et al. measured TF in 66 field sampling materials, identifying high fluorine levels at an LOD of 556 ppm (0.25 μg F/cm^2^) [51]. PIGE-measured TF indicated that the majority of fluorine remained non-extractable and was excluded from targeted analyses via GC-MS/MS, LC-MS/MS, and TOP assay, demonstrating that targeted methods are insufficient to close the fluorine mass balance within analytical error [50].

LIBS is emerging as a valuable surface-sensitive technique for characterizing fluoropolymers and detecting fluorine contamination. LIBS employs ultrashort laser pulses to ablate the sample and generate a plasma, whose optical emission reveals the elemental composition. Fluorine content is measured spectroscopically from the radiative relaxation of excited fluorine atoms (generally 685.6 nm, 703.7 nm and 775.5 nm); this technique has been used to determine fluorine content of chlorofluorocarbons in air using a flow-through manifold as low as 38 ppm [52]. Recent studies demonstrate the utility of LIBS for classifying fluoropolymer wastes with 81–100% accuracy, and quantifying fluorine contamination in consumer products with detection limits of approximately 160 ppm [53, 54]. Despite these advantages, LIBS cannot differentiate among fluorine-containing molecular species, limiting its use to TF determination rather than providing chemical speciation.

HR-CS-MAS likewise enables high-sensitivity analysis of TF in liquid and solid samples through vaporization or combustion in a graphite furnace to release the fluorine content [55]. Direct analysis of surface water by HR-CS-MAS following filtration (0.45 μm) yielded TF levels (148–270 ppb) in agreement with CIC analysis, with detection and quantitation limits of 0.8 and 2.7 ppb F^−^, respectively. However, HR-CS-MAS outperformed CIC in speed, precision, and sensitivity [56]. Yet, unlike CIC, HR-CS-MAS is not commercially implemented and is limited to the determination of TF content.

While the instrumental methods described above directly measure TF content in samples, the TOP assay offers a different approach for estimating TF: it employs chemical oxidation to quantify oxidizable PFAS precursors, followed by targeted LC-MS/MS analysis of transformation products [57, 58]. The TOP assay is a widely used laboratory method designed to indirectly quantify “precursor” compounds of PFAS that are not typically detected through routine targeted PFAS analysis [58]. The motivation behind the TOP assay comes from the understanding that many PFAS, especially polyfluorinated compounds, can degrade in the environment to form stable, recalcitrant perfluoroalkyl acids (PFAAs) [59]. The TOP assay provides a more complete picture of potential PFAS contamination by converting these “hidden” precursors into measurable PFAAs via strong oxidative treatment, usually using persulfate with heat or UV activation [59]. By comparing PFAA concentrations in a sample before and after oxidation, the presence and approximate amount of oxidizable precursors can be determined. This method is widely applied in various matrices, including water, soil [60], AFFF [61], or other industrial sources of PFAS [62].

Recent advancements have strengthened the TOP assay by improving oxidation efficiency and expanding detection of resulting PFAAs [24, 58, 63]; updated protocols include ultrashort-chain PFCAs such as TFA to capture small degradation products often missed by standard targeted lists [64], incorporate internal standards to track oxidation efficiency and matrix effects [63], and use modified procedures that separate charged and neutral polyfluoroalkyl substances prior to oxidation to enable more refined precursor quantification [65].

Achieving a complete fluorine mass balance using the TOP assay is challenging due to its limitations: it does not capture all PFAS precursors since only polyfluorinated compounds with at least one non-fluorinated carbon are intended to fully oxidize to PFAAs, terminal PFAAs (e.g., PFOS, PFOA) remain unchanged, and the assay cannot provide precursor speciation or mechanistic insight [58]. The detection of certain precursors in both pre- and post-TOP assay samples may indicate incomplete oxidation potentially due to oxidant consumption by matrix co-contaminants, ultimately leading to an underestimation of total precursor concentrations [58].

Given these limitations, the TOP assay, though valuable, should not be considered a direct TOF measurement [63]. Therefore it is more appropriate to treat it as an estimate of the “oxidizable precursor-derived PFAA burden.” For comprehensive fluorine mass balance assessments, complementary techniques, such as EOF analysis or combustion-based TF measurements, are needed to quantify the broader pool of fluorinated species beyond those amenable to oxidative conversion.

In summary, these techniques are highly complementary for fluorine analysis and by strategically combining these methods, researchers can overcome individual limitations and gain a comprehensive understanding of fluorine content, its chemical forms, and its distribution within various sample matrices, leading to more accurate and detailed insights into fluorine’s environmental and industrial roles.

## Expanding beyond conventional targeted analysis: challenges and advances in comprehensive detection of PFAS and other OFs

Most targeted LC–MS/MS approaches reported in the literature focus on the analysis of well-known PFAAs, including PFCAs and PFSAs, with chain lengths generally ranging from C_4_ to C_15_. However, a substantial fraction of the PFAS universe remains untargeted, and several factors contribute to this analytical gap, including regulatory definitions and classification boundaries [66], the immense chemical diversity and complexity [66], the lack of certified reference standards for most novel or lesser-studied PFAS [13], and extraction limitations [13, 67]. In addition, while LC-MS/MS method can quantify short-chain PFAS and certain neutral ether PFAS, conventional reversed-phase C18 methods are primarily optimized for ionic PFAS and offer limited retention for ultrashort-chain PFAS. Notably, neutral PFAS and polymeric OFs exhibit very low ionization efficiencies in LC-MS/MS that employs electrospray ionization (ESI), making them challenging to detect at low concentrations [13]. Recognizing and quantifying these non-target compounds is essential, including volatile fluorinated compounds, OFs that ionize exclusively in positive mode, compounds not retained in conventional SPE, and species not efficiently ionized under ESI, all of which are typically overlooked by conventional targeted analytical methods.

### Volatile fluorinated compounds

GC-MS-based methods are essential for characterizing volatile PFAS, including FTOHs, perfluoroalkane sulfonamides, and sulfonamido alcohols. Although non-volatile PFCAs can be derivatized to enable GC-MS analysis, most derivatization reagents do not react with the sulfonic acid groups of PFSAs under the same conditions used for carboxylic acids.

Element-specific GC detectors can complement GC–MS by providing direct evidence that chromatographic peaks contain fluorine. For example, GC–AED and fluorine-selective plasma emission detectors generate element-specific chromatograms that highlight fluorinated features within complex sample matrices. De Vega et al. [13] demonstrated the utility of GC-AED for detecting volatile OF compounds in ski wax extracts. Further, Gruber et al. [68] used a plasma emission detector with an echelle spectrometer in combination with GC to analyze the emission of unknown fluorinated substances from consumer products and food contact materials. These detectors are valuable for screening and prioritization of unknown OFs for further analysis. However, because they provide limited structural information, GC–MS remains necessary for confident molecular identification and quantification.

The performance of GC-based methods has been further improved through advanced sample-introduction techniques, such as thermal desorption (TD) [69], dynamic headspace (DHS) [16, 69], or solid-phase microextraction (SPME) [70], enabling low detection limits for volatile PFAS. TD is one such approach in which samples are heated to release adsorbed analytes prior to GC analysis, resulting in improved sensitivity. TD enables full automation and is well-suited for both routine monitoring and large-scale field studies [71]. It also supports both targeted quantification and non-targeted screening and is particularly effective for measuring volatile and semi-volatile OF compounds, including FTOHs (Table 2). Habib et al. demonstrated the utility of TD-GC-MS by coupling it with stir-bar sorptive extraction to quantify PFCAs in drinking water and wastewater, achieving detection limits ranging from 21.2 to 74.0 ppt [72]. Another approach that introduces samples into the GC is based on headspace (HS) techniques, which capture compounds that partition into the vapor phase above solid or liquid samples. A variation of this sample introduction technique is the dynamic headspace (DHS; purge-and-trap), which continuously purges the headspace, concentrating volatilized analytes onto an adsorbent that can then be introduced directly into the GC via TD for enhanced sensitivity. Recent applications of DHS-TD have demonstrated its utility for (semi-)volatile PFAS, including FTOHs, fluorotelomer acrylates, and sulfonamide-based derivatives. Application of the DHS-TD method in soil analysis has achieved detection limits as low as 7.02 ppt [73], and analogous approaches for aqueous matrices have reached detection limits of 2.17 ppt [74]. These developments enable highly sensitive, targeted quantification while also supporting broader, non-targeted screening for environmental monitoring.

**Table 2:** Overview of analytical techniques for detecting FTOHs across various matrices

Technique	Matrix	Ionization	Detected FTOHs	LOD	References
**DHS-TD-GC-HRMS**	Soil	Electron ionization (EI)	4:2 FTOH 6:2 FTOH 8:2 FTOH 10:2 FTOH	7.02–35.96 ppt	[73]
**DHS-TD-GCxGC-HRMS**	Water samples	EI	4:2 FTOH 6:2 FTOH 8:2 FTOH 10:2 FTOH	2.17–15.23 ppt	[74]
**TD-GC**-**MS/MS**	Indoor air	EI	4:2 FTOH 6:2 FTOH 8:2 FTOH 10:2 FTOH	0.2–0.7 ppt	[96]
**TD-GC-MS/MS**	Soil gas, sewer gas	EI	4:2 FTOH 5:2 FTOH 6:2 FTOH 7:2 FTOH 8:2 FTOH 10:2 FTOH 12:2 FTOH	0.03–9.8 ppt	[97]
**SPME-GC**/**MS**	Air and solid samples	EI	4:2 FTOH 6:2 FTOH 8:2 FTOH 10:2 FTOH	2.4–9.0 ppb (air) 13–37 ppb (solids)	[98]
**HS-SPME-GC**/**MS**	River/tap water and sediments	EI	6:2 FTOH 8:2 FTOH 10:2 FTOH	15–30 ppt (water) 0.3 ppb (sediment)	[78]
**APCI-UHPLC-MS/MS**	River water	APCI	4:2 FTOH 6:2 FTOH 8:2 FTOH 10:2 FTOH 7-Me-6:2 FTOH	0.7–75 ppb	[86]
**APCI-UHPLC-MS/MS**	River water	APCI	4:2 FTOH	0.003–6 ppb	[87]
**GC-APCI-MS/MS**	River water/wastewater	APCI	4:2 FTOH 6:2 FTOH 8:2 FTOH 10:2 FTOH	0.025–0.125 ppt	[85]
**APPI-HRMS**	Waterproof sprays	APPI	6:2 FTOH 8:2 FTOH 10:2 FTOH 7-Me-6:2 FTOH	6–315 ppb	[89]
**AP**P**I-UHPLC-MS/MS**	River water	APPI	4:2 FTOH 6:2 FTOH 8:2 FTOH 10:2 FTOH 7-Me-6:2 FTOH	0.08–1 ppb*	[86]
**GC-APPI-HRMS**	River water/tap water	APPI	4:2 FTOH 6:2 FTOH 8:2 FTOH 10:2 FTOH 7-Me-6:2 FTOH	0.02–0.06 ppt	[91]

SPME is a solvent-free technique that integrates sampling, extraction, and sample introduction in a single device, which uses a sorbent-coated fiber exposed directly to a sample (direct-immersion SPME) or its headspace (HS-SPME) to concentrate trace analytes, which are then thermally desorbed for GC-MS analysis [70]. SPME is easily automated, environmentally friendly, and widely applied in environmental monitoring [75]. In particular, HS-SPME mode is effective for isolating volatile compounds from complex matrices [76]. SPME-GC-MS has been increasingly used for the analysis of volatile PFAS. For example, Alzaga and Bayona [77] used SPME-GC-MS to detect PFCAs in environmental waters with in-port derivatization, where the non-volatile PFCAs were chemically derivatized in the injector to form volatile derivatives suitable for GC analysis at LODs of 0.02–2.5 ppb and demonstrating its utility for PFAS analysis in aquatic environments. Similarly, Bach et al. [78] used HS-SPME-GC-MS for the simultaneous determination of perfluoroalkyl iodides, sulfonamides, FTOHs, and other volatile PFAS (e.g., fluorotelomer iodides and fluorotelomer acrylates/methacrylates) in surface water (LOD: 6–30 ppt) and in sediments (LOD: 0.3–0.9 ppb). Monteleone et al. [79] also used HS-SPME-GC-MS/MS for the detection of PFCAs in river water samples (LOD: 0.08–6.6 ppt); however, the approach required prior derivatization to convert PFCAs into GC-amenable derivatives prior to HS-SPME.

### Fluorinated compounds exclusively ionizing in ESI positive mode

**Fig. 6:**
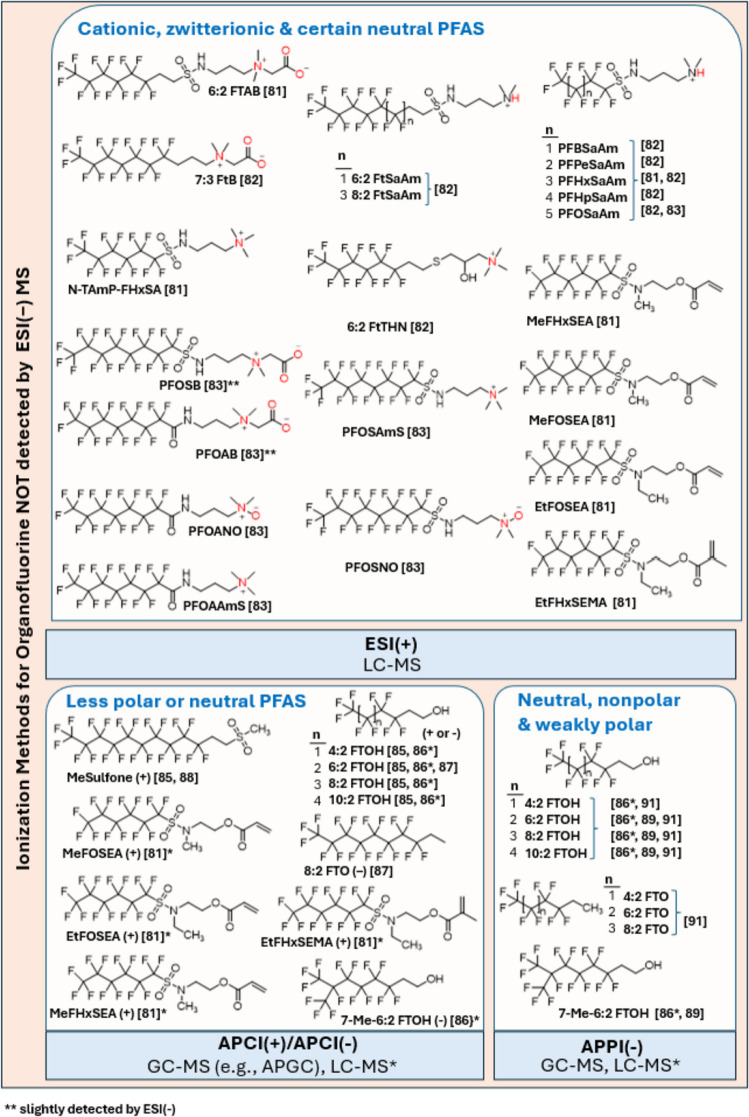
Representative classes of PFAS that are detectable by alternative ionization techniques—including electrospray ionization in positive mode (+ESI), atmospheric pressure chemical ionization in positive or negative mode (+/-APCI), and atmospheric pressure photoionization in negative mode (-APPI)—but are commonly missed or inefficiently ionized under electrospray ionization in negative mode (-ESI). Shown structures include fluorotelomer amido betaine (FTAB), fluorotelomer betaine (FtB), fluorotelomer sulfonamidoamines (FtSaAm), N-trimethylaminopropyl perfluorohexane sulfonamide (N-TAFmP-FHxSA), fluorotelomer thioether amine (FtTHN), perfluorooctane sulfonamido betaine (PFOSB), perfluorooctane amido betaine (PFOAB), perfluorooctane amido nitrile (PFOANO), perfluorooctane sulfonamido amine homologues including PFBSaAm, PFPeSaAm, PFHxSaAm, PFHpSaAm, and PFOSaAm, perfluorooctane amido amines (PFOAAmS), perfluorooctane sulfonamido amines (PFOSAmS), and perfluorooctane sulfonamido oxime (PFOSNO), as well as sulfonamido ether and methacrylate derivatives including methyl perfluorohexane sulfonamide ethyl ether (MeFHxSEA), methyl perfluorooctane sulfonamide ethyl ether (MeFOSEA), ethyl perfluorooctane sulfonamide ethyl ether (EtFOSEA), and ethyl perfluorohexane sulfonamido methacrylate (EtFHxSEMA). Neutral and weakly polar PFAS detectable by APCI or APPI include fluorosulfones such as methyl fluorosulfone (MeSulfone), fluorotelomer alcohols (FTOHs), fluorotelomer olefins (FTOs), and neutral sulfonamido ethers such as MeFOSEA, EtFOSEA, and MeFHxSEA. Overall, this figure highlights structurally diverse PFAS classes that evade detection under standard ESI^−^ but become accessible when using orthogonal ionization techniques

Negative electrospray ionization (-ESI) mode is commonly used in PFAS LC-MS/MS analysis. However, this approach overlooks many other PFAS and OF classes, including cationic, zwitterionic, and certain neutral compounds (Fig. 6), creating significant gaps in fluorine mass balance coverage [80]. Research has shown that using positive mode (+)ESI can uncover significant amounts of OF compounds that would otherwise go undetected, thereby improving the PFAS mass balance by aligning TOF levels with structurally identified compounds [81]. For instance, in contaminated soils and sediments from fire-training area source zones, cationic and zwitterionic PFAS accounted for up to 97% of the total PFAS mass—an insight made possible only through positive mode LC-HRMS analysis [80]. Backe et al. [82] detected zwitterionic, cationic, and some newly identified PFAS including fluorotelomer thiohydroxy ammonium, fluorotelomer sulfonamido amines, and perfluoroalkyl sulfonamido amines, in AFFF formulations and groundwater samples in + ESI mode [82]. Similarly, a survey of river sediments across France identified fluorotelomer sulfonamide betaines and amines (Fig. 6), two classes largely undetectable by -ESI, at concentrations approaching 10X those of PFOS in some sites [83]. Comprehensive LC-HRMS analyses of AFFF-impacted soils, conducted in both + ESI and -ESI modes, revealed that zwitterionic and cationic PFAS (Fig. 6) constituted the dominant share (up to 97% mass) of the total PFAS burden, thereby closing critical detection gaps [80].

### Alternative ionization

ESI is widely employed in PFAS analysis for its effectiveness in ionizing polar compounds. However, it falls short in detecting neutral, nonpolar, volatile, and certain emerging PFAS. Some PFAS precursor compounds that are poorly ionized by conventional ESI in both positive and negative modes, necessitate derivatization to enable their effective inclusion in ESI-MS workflows [84]. As a result, ESI-based methods are likely to capture only part of the TOF and could lead to an underestimation of overall OF contamination. Alternative ionization techniques complement ESI by offering distinct ionization mechanisms capable of efficiently targeting PFAS classes poorly ionized by ESI. Prominent alternative approaches include atmospheric pressure chemical ionization (APCI) and atmospheric photoionization (APPI).

APCI is a soft ionization technique that enables the selective and sensitive detection of less polar or neutral PFAS that are inefficiently ionized by ESI [85]. APCI exhibits high sensitivity and low susceptibility to matrix effects in complex environmental samples, supporting robust quantification and broad linear dynamic ranges. It also preserves molecular ions with limited fragmentation, improving structural specificity for diverse or unknown PFAS [85]. A study has shown that its use in river and wastewater matrices has not only improved the detection (LOD: 0.025–0.125 ppt or 1–5 fg) of known volatile and semi-volatile PFAS such as FTOHs (Table 2) but has also facilitated the discovery of novel PFAS analogues such as methyl-sulfone derivatives [85]. Another study reported that APCI provides improved ionization efficiency and selectivity for semi-volatile PFAS, including FTOH, FOSA, and FOSE, with LODs of 0.7 to 75 ppb, compared to ESI-based approaches [86]. Ayala-Cabrera et al. [87] reported that the use of ultra-high-performance liquid chromatography (UHPLC)–MS/MS equipped with APCI enabled the detection of low concentrations of neutral PFASs, including fluorotelomer olefins (FTOs) and FTOHs (Fig. 6). Lauria et al. used APCI-GC-IMS to analyze killer whale blubber samples and detected fluorotelomer sulfones at LOQs of 0.08–0.29 ppb [88].

APPI is a valuable ionization technique for MS analysis of PFAS, particularly those that are neutral, non-polar, or weakly polar and poorly ionized by traditional ESI [89] (Fig. 6). APPI uses vacuum ultraviolet photons (∼10 eV) from a krypton lamp to photoionize analytes or dopants, efficiently generating molecular and radical ions with minimal fragmentation, which expands the range of detectable PFAS for comprehensive PFAS profiling [89, 90]. ESI and APCI are constrained by their reliance on ionic or moderately polar analytes, whereas APPI—particularly with dopant assistance—can effectively ionize compounds across a wider range of structures and polarities [90]. It is also less affected by ion suppression and salt interference than APCI or ESI, enhancing reliability in complex matrices. Ayala-Cabrera et al. developed a GC-APPI-HRMS method to detect neutral PFAS including FTOs, FTOHs, FOSAs and FOSEs in river water samples with low LODs (0.02–15 ppt) [91]. Table 2 summarizes the techniques that are used to analyze FTOHs. Together, these techniques expand the analytical scope for identifying both known and emerging PFAS and related OFs in environmental samples.

Beyond APCI and APPI, additional emerging ionization strategies have been reported for OFs in environmental and biological matrices, particularly ambient/direct approaches that enable rapid screening with little or no chromatographic separation. Some examples include dielectric barrier discharge ionization [92], matrix-assisted laser desorption [93], paper spray mass spectrometry [94], and surface-assisted laser desorption ionization [95].

### Non-retained OF in SPE

Conventional SPE methods are effective for many PFAS but struggle with certain classes, including ultrashort-chain and short-chain PFAS. Additionally, when using WAX sorbents, neutral PFAS such as FTOHs often exhibit limited recovery [99]. Several alternative preconcentration techniques have been developed to improve PFAS extraction recovery and detection. For instance, the large-volume injection technique introduces a significantly larger sample volume directly into the instrument, bypassing the time-consuming SPE step. The large volume injection approach drastically lowers LOD and improves sensitivity for trace contaminants. However, this approach is limited to aqueous samples, is less suitable for long-chain PFAS, and presents specific challenges for dirty samples due to column fouling and matrix effects [100]. Recent developments in SPME, such as multi-monolithic fiber SPME incorporating fluorophilic and anion-exchange functionalities, have shown effective enrichment of short-chain PFAS from small sample volumes [101]. Additional advancements in PFAS preconcentration include the development of novel adsorbent materials, such as fluorinated covalent organic frameworks [102, 103], which exhibit broad PFAS retention capabilities and improved efficiency for short-chain compounds [101]. Affinity-based extraction using proteins such as bovine serum albumin has also emerged as a selective and efficient approach for PFAS analysis [101]. Other emerging techniques, such as rapid electrochemical aerosol formation, have also shown promise, enabling the concentration of OF by several orders of magnitude within a short time frame [104].

## Summary, recommendations, and future work

Recent advances continue to reveal substantial gaps in our ability to detect and characterize PFAS and other OF across environmental and biological systems. Conventional targeted methods, most notably EPA Method 1633, capture only a small fraction of the thousands of fluorinated chemicals in use and routinely underestimate total PFAS. As a result, key classes such as ultrashort-chain PFAS, neutral and lipophilic PFAS, fluorinated pharmaceuticals and pesticides, and volatile PFAS are frequently undetected. These gaps arise from analyte losses during extraction and ionization, poor detection limits, a lack of reference standards, and the narrow chemical scope of current validated methods. Consequently, fluorine mass-balance studies consistently show that targeted PFAS account for only a small portion of EOF or AOF. Although complementary strategies such as the TOP assay combined with targeted LC–MS/MS, suspect screening, and non-target HRMS can reveal precursor contributions, the TOP assay does not capture the full OF pool, as many compounds do not oxidize to the PFAS end-products quantified by LC-MS/MS. Together, these limitations highlight the need for more comprehensive analytical frameworks that can capture the breadth of OF chemistries.

Analytical tools are expanding to address these shortcomings. GC–MS is increasingly applied, particularly when paired with TD, HS sampling, or SPME, to quantify volatile and neutral PFAS that escape LC–MS/MS detection. Alternative ionization sources such as APCI and APPI further extend coverage to zwitterionic, cationic, and weakly ionizable compounds. At the same time, persistent losses of non-retained OF during SPE have led to the development of new sorbents, protein-affinity approaches, and advanced SPME formats designed to retain a wider range of fluorinated species. These innovations underscore the urgent need for multi-technique workflows to close the fluorine mass-balance gap.

Achieving meaningful progress will require coordinated development of fluorine-retentive sample-preparation strategies and integrated analytical approaches. Techniques such as ^19^F-NMR, CIC, and non-targeted MS acquisition should be incorporated into TF measurements to capture the full spectrum of fluorinated compounds. These measurements can then be paired with methods that provide chemical speciation: XPS offers spatially resolved surface information for solid matrices, while solid-state ^19^F-NMR provides complementary molecular-level characterization. For solids and heterogeneous materials, TF methods such as CIC can guide the design of extraction and cleanup protocols optimized for high fluorine recovery.

## Data Availability

The authors confirm that the data supporting the findings of this study are available within the article.

## Abbreviations

PFAS: Per- and polyfluoroalkyl substances
OECD: Organization for Economic Co-operation and Development
US EPA: US Environmental Protection Agency
OF: Organofluorine
IF: Inorganic fluorine
AOF: Adsorbable organofluorine
EOF: Extractable organofluorine
N-EOF: Non-extractable organofluorine
TF: Total fluorine/fluoride
TOF: Total organofluorine
LC-MS/MS: Liquid chromatography-tandem mass spectrometry
LC-HRMS: Liquid chromatography-high resolution mass spectrometry
PFOA: Perfluorooctanoic acid
PFOS: Perfluorooctane sulfonate
CIC: Combustion ion chromatography
^19^F-NMR: Fluorine-19 nuclear magnetic resonance
PIGE: Particle-induced gamma-ray emission
XPS: X-ray photoelectron spectroscopy
SPE: Solid-phase extraction
IMS: Ion mobility spectrometry
PFCA: Perfluoroalkyl carboxylic acids
PFSA: Perfluoroalkyl sulfonic acids
PFEA: Perfluoroether acids
PFPA: Perfluoroalkyl phosphonic acids
PFPIA: Perfluoroalkyl phosphinic acid
PFAA: Perfluoroalkyl acids
FTOH: Fluorotelomer alcohol
TFA: Trifluoroacetic acid
GC-MS: Gas chromatography-mass spectrometry
AED: Atomic emission detection
TOP: Total oxidizable precursor
GAC: Granular activated carbon
LOD: Limit of detection
SSFP: Steady-state free precession
SS: Solid-state
CRAFT: Complete reduction to amplitude frequency tables
DNP: Dynamic nuclear polarization
LIBS: Laser-induced breakdown spectroscopy
HR-CS-MAS: High-resolution continuum-source molecular absorption spectroscopy
AFFF: Aqueous film-forming foam
ESI: Electrospray ionization
APCI: Atmospheric pressure chemical ionization
APPI: Atmospheric pressure photoionization
UHPLC: Ultra-high-performance liquid chromatography
TD: Thermal desorption
DHS: Dynamic headspace
SPME: Solid-phase microextraction
HS: Headspace
EI: Electron ionization

## References

[1] Han J, Kiss L, Mei H, Remete AM, Ponikvar-Svet M, Sedgwick DM, et al. Chemical aspects of human and environmental overload with fluorine. Chem Rev. 2021;121(8):4678–742. 10.1021/acs.chemrev.0c01263. 33723999 10.1021/acs.chemrev.0c01263 PMC8945431

[2] Krause M, Stoesser J, de Carvalho AR, Hanozin E, Jacobs G, Voorspoels S, et al. Analysis of needs for enforcement of PFAS in articles and chemical products: Nordic Council of Ministers; 2024. Available from: https://pub.norden.org/temanord2024-510. Accessed 09 Mar 2026.

[3] USEPA. TSCA Section 8(a)(7) Reporting and Recordkeeping Requirements for Perfluoroalkyl and Polyfluoroalkyl Substances 2025. Available from: https://www.epa.gov/assessing-and-managing-chemicals-under-tsca/tsca-section-8a7-reporting-and-recordkeeping. Accessed 18 Sep 2025.

[4] OECD. Reconciling terminology of the universe of per-and polyfluoroalky l substances: recommendations and practical guidance. Ser Risk Manag. 2021;61:1–43.

[5] Wang Z, Buser AM, Cousins IT, Demattio S, Drost W, Johansson O, et al. A new OECD definition for per-and polyfluoroalkyl substances. Environ Sci Technol. 2021;55(23):15575–8. 10.1021/acs.est.1c06896. 34751569 10.1021/acs.est.1c06896

[6] Evich MG, Davis MJ, McCord JP, Acrey B, Awkerman JA, Knappe DR, et al. Per-and polyfluoroalkyl substances in the environment. Science. 2022;375(6580):eabg9065. 35113710 10.1126/science.abg9065 PMC8902460

[7] USEPA. Comp Tox Chemicals Dashboard 2024. Available from: https://comptox.epa.gov/dashboard/chemical-lists/PFASSTRUCT. Accessed 10 Aug 2026.

[8] USEPA. Final PFAS national primary drinking water regulation. 2024. Available from: https://www.epa.gov/sdwa/and-polyfluoroalkylsubstances-pfas. Accessed 08 Mar 2026.

[9] Buck RC, Franklin J, Berger U, Conder JM, Cousins IT, De Voogt P, et al. Perfluoroalkyl and polyfluoroalkyl substances in the environment: terminology, classification, and origins. Integr Environ Assess Manag. 2011;7(4):513–41. 10.1002/ieam.258. 21793199 10.1002/ieam.258 PMC3214619

[10] Ruyle BJ, Pennoyer EH, Vojta S, Becanova J, Islam M, Webster TF, et al. High organofluorine concentrations in municipal wastewater affect downstream drinking water supplies for millions of Americans. Proc Natl Acad Sci U S A. 2025;122(3):e2417156122. 10.1073/pnas.2417156122. 39761386 10.1073/pnas.2417156122 PMC11761303

[11] Aro R, Eriksson U, Kärrman A, Jakobsson K, Yeung LW. Extractable organofluorine analysis: a way to screen for elevated per-and polyfluoroalkyl substance contamination in humans? Environ Int. 2022;159:107035. 10.1016/j.envint.2021.107035. 34896670 10.1016/j.envint.2021.107035

[12] Aro R, Carlsson P, Vogelsang C, Kärrman A, Yeung LW. Fluorine mass balance analysis of selected environmental samples from Norway. Chemosphere. 2021;283:131200. 10.1016/j.chemosphere.2021.131200. 34157625 10.1016/j.chemosphere.2021.131200

[13] de Vega RG, Plassmann M, Clases D, Zangger K, Müller V, Rosenberg E, et al. A multi-platform approach for the comprehensive analysis of per-and polyfluoroalkyl substances (PFAS) and fluorine mass balance in commercial ski wax products. Anal Chim Acta. 2024;1314:342754. 10.1016/j.aca.2024.342754. 38876512 10.1016/j.aca.2024.342754

[14] Baqar M, Chen H, Yao Y, Sun H. Latest trends in the environmental analysis of PFAS including nontarget analysis and EOF-, AOF-, and TOP-based methodologies. Anal Bioanal Chem. 2025;417(3):555–71. 10.1007/s00216-024-05643-9. 39570388 10.1007/s00216-024-05643-9

[15] Koch A, Aro R, Wang T, Yeung LW. Towards a comprehensive analytical workflow for the chemical characterisation of organofluorine in consumer products and environmental samples. TRAC Trends Anal Chem. 2020;123:115423. 10.1016/j.trac.2019.02.024.

[16] Shen Y, Wang L, Ding Y, Liu S, Li Y, Zhou Z, et al. Trends in the analysis and exploration of per-and polyfluoroalkyl substances (PFAS) in environmental matrices: a review. Crit Rev Anal Chem. 2024;54(8):3171–95. 10.1080/10408347.2023.2231535. 37410543 10.1080/10408347.2023.2231535

[17] Teymoorian T, Munoz G, Vo Duy S, Liu J, Sauvé S. Tracking PFAS in drinking water: a review of analytical methods and worldwide occurrence trends in tap water and bottled water. ACS ES&T Water. 2023;3(2):246–61. 10.1021/acsestwater.2c00387.

[18] Smith SJ, Lauria M, Higgins CP, Pennell KD, Blotevogel J, Arp HPH. The need to include a fluorine mass balance in the development of effective technologies for PFAS destruction. Environ Sci Technol. 2024;58(6):2587–90. 10.1021/acs.est.3c10617. 38314573 10.1021/acs.est.3c10617 PMC10867837

[19] Aguilar JMN, Wijayahena MK, Running LS, Phatthanawiwat K, Choodum A, Wallace JS, et al. Comprehensive evaluation of the analytical efficiency of combustion ion chromatography for per-and polyfluoroalkyl substances in biological and environmental matrices. Environ Sci Technol. 2025;59(6):3218–28. 10.1021/acs.est.4c12443. 39913527 10.1021/acs.est.4c12443 PMC13104706

[20] Cioni L, Nikiforov V, Benskin JP, Coêlho ACM, Dudasova S, Lauria MZ, et al. Combining advanced analytical methodologies to uncover suspect PFAS and fluorinated pharmaceutical contributions to extractable organic fluorine in human serum (Tromsø Study). Environ Sci Technol. 2024;58(29):12943–53. 10.1021/acs.est.4c03758. 38985529 10.1021/acs.est.4c03758 PMC11271008

[21] Pan Y, Helbling DE. Revealing the factors resulting in incomplete recovery of perfluoroalkyl acids (PFAAs) when implementing the adsorbable and extractable organic fluorine methods. Water Res. 2023;244:120497. 10.1016/j.watres.2023.120497. 37619306 10.1016/j.watres.2023.120497

[22] Han Y, Pulikkal VF, Sun M. Comprehensive validation of the adsorbable organic fluorine analysis and performance comparison of current methods for total per-and polyfluoroalkyl substances in water samples. ACS ES&T Water. 2021;1(6):1474–82. 10.1021/acsestwater.1c00047.

[23] Miyake Y, Yamashita N, So MK, Rostkowski P, Taniyasu S, Lam PK, et al. Trace analysis of total fluorine in human blood using combustion ion chromatography for fluorine: a mass balance approach for the determination of known and unknown organofluorine compounds. J Chromatogr A. 2007;1154(1–2):214–21. 10.1016/j.chroma.2007.03.084. 17416376 10.1016/j.chroma.2007.03.084

[24] Cioni L, Plassmann M, Benskin JP, Coelho ACM, Nøst TH, Rylander C, et al. Fluorine mass balance, including total fluorine, extractable organic fluorine, oxidizable precursors, and target per-and polyfluoroalkyl substances, in pooled human serum from the Tromsø population in 1986, 2007, and 2015. Environ Sci Technol. 2023;57(40):14849–60. 10.1021/acs.est.3c03655. 37747946 10.1021/acs.est.3c03655 PMC10569050

[25] Shard AG. Detection limits in XPS for more than 6000 binary systems using Al and Mg Kα X‐rays. Surf Interface Anal. 2014;46(3):175–85. 10.1002/sia.5406.

[26] Lincoln SA, Jahn KL, Schallenberger JR, Saffer DM, Freeman KH, inventors; The Penn State Research Foundation, assignee. Screening/Analysis of Fluorocarbons using X-ray Photoelectron Spectroscopy. United States patent application US 17/783,613. 2022. Available from: https://patents.google.com/patent/US20220373486A1/en. Accessed 09 March 2026.

[27] Tighe M, Jin Y, Whitehead HD, Hayes K, Lieberman M, Pannu M, et al. Screening for per-and polyfluoroalkyl substances in water with particle induced gamma-ray emission spectroscopy. ACS ES&T Water. 2021;1(12):2477–84. 10.1021/acsestwater.1c00215.

[28] Moody CA, Kwan WC, Martin JW, Muir DC, Mabury SA. Determination of perfluorinated surfactants in surface water samples by two independent analytical techniques: liquid chromatography/tandem mass spectrometry and ^19^F NMR. Anal Chem. 2001;73(10):2200–6. 10.1021/ac0100648. 11393841 10.1021/ac0100648

[29] Gauthier JR, Kock F, Downey K, Moraes T, Almeida LS, Muir DC, et al. Steady state free precession NMR without fourier transform: redefining the capabilities of ^19^F NMR as a discovery tool. Angew Chem. 2025;137(15):e202422971. 10.1002/ange.202422971. 10.1002/anie.202422971 PMC11976210 39874133

[30] Ronda K, Gauthier J, Singaravadivel K, Costa PM, Downey K, Wolff WW, et al. NMR as a discovery tool: exploration of industrial effluents discharged into the environment. Magn Reson Chem. 2025;63(7):453–75. 10.1002/mrc.5527. 40360259 10.1002/mrc.5527 PMC12129648

[31] Faber KA, Pomerantz WC, Gray JL, Hubbard LE, Kolpin DW, Arnold WA. Revealing organofluorine contamination in effluents and surface waters with complementary analytical approaches: fluorine-19 nuclear magnetic resonance spectroscopy (19F-NMR) and liquid chromatography-tandem mass spectrometry (LC-MS/MS). Environ Sci Technol. 2025;59(28):14695–706. 10.1021/acs.est.5c05079. 40629832 10.1021/acs.est.5c05079

[32] Balasuryia D, Queral-Beltran A, Vick T, Simpson S, Lacorte S, Aga DS, et al. Experimental determination of p K a for 10 PFAS, mono-, di-, and trifluoroacetic acid by 19F-NMR. Environ Sci Technol Lett. 2025. 10.1021/acs.estlett.5c00688. 40951867 10.1021/acs.estlett.5c00688 PMC12424170

[33] Forster AL, Zhang Y, Westerman DC, Richardson SD. Improved total organic fluorine methods for more comprehensive measurement of PFAS in industrial wastewater, river water, and air. Water Res. 2023;235:119859. 10.1016/j.watres.2023.119859. 36958221 10.1016/j.watres.2023.119859

[34] Camdzic D, Dickman RA, Aga DS. Total and class-specific analysis of per-and polyfluoroalkyl substances in environmental samples using nuclear magnetic resonance spectroscopy. J Hazard Mater Lett. 2021;2:100023. 10.1016/j.hazl.2021.100023.

[35] Camdzic D, Dickman RA, Joyce AS, Wallace JS, Ferguson PL, Aga DS. Quantitation of total PFAS including trifluoroacetic acid with fluorine nuclear magnetic resonance spectroscopy. Anal Chem. 2023;95(13):5484–8. 10.1021/acs.analchem.2c05354. 36946571 10.1021/acs.analchem.2c05354 PMC10601338

[36] Gauthier JR, Mabury SA. Identifying unknown fluorine-containing compounds in environmental samples using 19F NMR and spectral database matching. Environ Sci Technol. 2023;57(23):8760–7. 10.1021/acs.est.3c01220. 37259970 10.1021/acs.est.3c01220

[37] Gauthier JR, Mabury SA. Noise-reduced quantitative fluorine NMR spectroscopy reveals the presence of additional per-and polyfluorinated alkyl substances in environmental and biological samples when compared with routine mass spectrometry methods. Anal Chem. 2022;94(7):3278–86. 10.1021/acs.analchem.1c05107. 35148065 10.1021/acs.analchem.1c05107

[38] Marsh RW, Kewalramani JA, de Souza BB, Meegoda JN. The use of a fluorine mass balance to demonstrate the mineralization of PFAS by high frequency and high power ultrasound. Chemosphere. 2024;352:141270. 10.1016/j.chemosphere.2024.141270. 38280651 10.1016/j.chemosphere.2024.141270

[39] Thijs M, Laletas E, Quinn CM, Raguraman SV, Carr B, Bierganns P. Total and class-specific determination of fluorinated compounds in consumer and food packaging samples using fluorine-19 solid-state nuclear magnetic resonance spectroscopy. Anal Chem. 2024;96(21):8282–90. 10.1021/acs.analchem.3c04404. 38717341 10.1021/acs.analchem.3c04404

[40] Bruker. Dynamic Nuclear Polarization NMR (DNP-NMR) 2026. Available from: https://www.bruker.com/pt/products-and-solutions/mr/nmr/dnp-nmr.html. Accessed 10 Mar 2026.

[41] Kassis CM, Steehler JK, Betts DE, Guan Z, Romack TJ, DeSimone JM, et al. XPS studies of fluorinated acrylate polymers and block copolymers with polystyrene. Macromolecules. 1996;29(9):3247–54. 10.1021/ma951782x.

[42] Tokranov AK, Nishizawa N, Amadei CA, Zenobio JE, Pickard HM, Allen JG, et al. How do we measure poly-and perfluoroalkyl substances (PFASs) at the surface of consumer products? Environ Sci Technol Lett. 2018;6(1):38–43. 10.1021/acs.estlett.8b00600. 33283017 10.1021/acs.estlett.8b00600 PMC7713715

[43] Lee AY, Powell CJ, Gorham JM, Morey A, Scott JHJ, Hanisch RJ. Development of the NIST X-ray photoelectron spectroscopy (XPS) database, version 5. Data Sci J. 2024;23(1). 10.5334/dsj-2024-045.

[44] Wu Y, Miller GZ, Gearhart J, Peaslee G, Venier M. Side-chain fluorotelomer-based polymers in children car seats. Environ Pollut. 2021;268:115477. 10.1016/j.envpol.2020.115477. 33221613 10.1016/j.envpol.2020.115477

[45] Lang JR, McDonough J, Guillette TC, Storch P, Anderson J, Liles D, et al. Characterization of per- and polyfluoroalkyl substances on fire suppression system piping and optimization of removal methods. Chemosphere. 2022;308:136254. 10.1016/j.chemosphere.2022.136254. 36108758 10.1016/j.chemosphere.2022.136254

[46] Rodrigo PM, Navarathna C, Pham MT, McClain SJ, Stokes S, Zhang X, et al. Batch and fixed bed sorption of low to moderate concentrations of aqueous per-and poly-fluoroalkyl substances (PFAS) on Douglas fir biochar and its Fe3O4 hybrids. Chemosphere. 2022;308:136155. 10.1016/j.chemosphere.2022.136155. 36099986 10.1016/j.chemosphere.2022.136155

[47] Mantripragada S, Deng D, Zhang L. Remediation of GenX from water by amidoxime surface-functionalized electrospun polyacrylonitrile nanofibrous adsorbent. Chemosphere. 2021;283:131235. 10.1016/j.chemosphere.2021.131235. 34153919 10.1016/j.chemosphere.2021.131235

[48] Ritter EE, Dickinson ME, Harron JP, Lunderberg DM, DeYoung PA, Robel AE, et al. PIGE as a screening tool for per-and polyfluorinated substances in papers and textiles. Nucl Instrum Methods Phys Res Sect B. 2017;407:47–54. 10.1016/j.nimb.2017.05.052.

[49] Whitehead HD, Venier M, Wu Y, Eastman E, Urbanik S, Diamond ML, et al. Fluorinated compounds in North American cosmetics. Environ Sci Technol Lett. 2021;8(7):538–44. 10.1021/acs.estlett.1c00240.

[50] Robel AE, Marshall K, Dickinson M, Lunderberg D, Butt C, Peaslee G, et al. Closing the mass balance on fluorine on papers and textiles. Environ Sci Technol. 2017;51(16):9022–32. 10.1021/acs.est.7b02080. 28712295 10.1021/acs.est.7b02080

[51] Rodowa AE, Christie E, Sedlak J, Peaslee GF, Bogdan D, DiGuiseppi B, et al. Field sampling materials unlikely source of contamination for perfluoroalkyl and polyfluoroalkyl substances in field samples. Environ Sci Technol Lett. 2020;7(3):156–63. 10.1021/acs.estlett.0c00036.

[52] Cremers DA, Radziemski LJ. Detection of chlorine and fluorine in air by laser-induced breakdown spectrometry. Anal Chem. 1983;55(8):1252–6. 10.1021/ac00259a017.

[53] Liu K, Tian D, Li C, Li Y, Yang G, Ding Y. A review of laser-induced breakdown spectroscopy for plastic analysis. TrAC Trends Anal Chem. 2019;110:327–34. 10.1016/j.trac.2018.11.025.

[54] Gondal MA, Habibullah YB, Oloore LE, Iqbal MA. Determination of carcinogenic fluorine in cigarettes using pulsed UV laser-induced breakdown spectroscopy. Appl Opt. 2015;54(17):5560–7. 10.1364/AO.54.005560. 26192861 10.1364/AO.54.005560

[55] Gehrenkemper L, Simon F, Roesch P, Fischer E, von der Au M, Pfeifer J, et al. Determination of organically bound fluorine sum parameters in river water samples—comparison of combustion ion chromatography (CIC) and high resolution-continuum source-graphite furnace molecular absorption spectrometry (HR-CS-GFMAS). Anal Bioanal Chem. 2021;413(1):103–15. 10.1007/s00216-020-03010-y. 33164152 10.1007/s00216-020-03010-y PMC8473383

[56] Simon F, Gehrenkemper L, von der Au M, Wittwer P, Roesch P, Pfeifer J, et al. A fast and simple PFAS extraction method utilizing HR–CS–GFMAS for soil samples. Chemosphere. 2022;295:133922. 10.1016/j.chemosphere.2022.133922. 35143867 10.1016/j.chemosphere.2022.133922

[57] Antell EH, Yi S, Olivares CI, Ruyle BJ, Kim JT, Tsou K, et al. The total oxidizable precursor (TOP) assay as a forensic tool for per-and polyfluoroalkyl substances (PFAS) source apportionment. ACS ES&T Water. 2023;4(3):948–57. 10.1021/acsestwater.3c00106.

[58] Ateia M, Chiang D, Cashman M, Acheson C. Total oxidizable precursor (TOP) assay─ best practices, capabilities and limitations for PFAS site investigation and remediation. Environ Sci Technol Lett. 2023;10(4):292–301. 10.1021/acs.estlett.3c00061. 37313434 10.1021/acs.estlett.3c00061 PMC10259459

[59] Houtz EF, Sedlak DL. Oxidative conversion as a means of detecting precursors to perfluoroalkyl acids in urban runoff. Environ Sci Technol. 2012;46(17):9342–9. 10.1021/es302274g. 22900587 10.1021/es302274g

[60] Wellmitz J, Bandow N, Koschorreck J. Long-term trend data for PFAS in soils from German ecosystems, including TOP assay. Sci Total Environ. 2023;893:164586. 10.1016/j.scitotenv.2023.164586. 37271402 10.1016/j.scitotenv.2023.164586

[61] Ruyle BJ, Thackray CP, McCord JP, Strynar MJ, Mauge-Lewis KA, Fenton SE, et al. Reconstructing the composition of per-and polyfluoroalkyl substances in contemporary aqueous film-forming foams. Environ Sci Technol Lett. 2020;8(1):59–65. 10.1021/acs.estlett.0c00798. 10.1021/acs.estlett.0c00798 PMC7898139 33628855

[62] Schellenberger S, Liagkouridis I, Awad R, Khan S, Plassmann M, Peters G, et al. An outdoor aging study to investigate the release of per-and polyfluoroalkyl substances (PFAS) from functional textiles. Environ Sci Technol. 2022;56(6):3471–9. 10.1021/acs.est.1c06812. 35213128 10.1021/acs.est.1c06812 PMC8928479

[63] Tsou K, Antell E, Duan Y, Olivares CI, Yi S, Alvarez-Cohen L, et al. Improved total oxidizable precursor assay for quantifying polyfluorinated compounds amenable to oxidative conversion to perfluoroalkyl carboxylic acids. ACS ES&T Water. 2023;3(9):2996–3003. 10.1021/acsestwater.3c00224.

[64] Janda J, Nödler K, Scheurer M, Happel O, Nürenberg G, Zwiener C, et al. Closing the gap–inclusion of ultrashort-chain perfluoroalkyl carboxylic acids in the total oxidizable precursor (TOP) assay protocol. Environ Sci Process Impacts. 2019;65(11):1926–35. 10.1039/C9EM00169G. 10.1039/c9em00169g 31183483

[65] Antell EH, Yi S, Olivares CI, Chaudhuri S, Ruyle BJ, Alvarez-Cohen L, et al. Selective quantification of charged and neutral polyfluoroalkyl substances using the total oxidizable precursor (TOP) assay. Environ Sci Technol. 2025;66(7):3780–91. 10.1021/acs.est.4c13837. 10.1021/acs.est.4c13837 PMC11866920 39946740

[66] Müller V, Kindness A, Feldmann J. Fluorine mass balance analysis of PFAS in communal waters at a wastewater plant from Austria. Water Res. 2023;244:120501. 10.1016/j.watres.2023.120501. 37647770 10.1016/j.watres.2023.120501

[67] Schultes L, Vestergren R, Volkova K, Westberg E, Jacobson T, Benskin JP. Per-and polyfluoroalkyl substances and fluorine mass balance in cosmetic products from the Swedish market: implications for environmental emissions and human exposure. Environ Sci Process Impacts. 2018;69(12):1680–90. 10.1039/C8EM00368H. 10.1039/c8em00368h 30427048

[68] Gruber L, Ewender J, Fiedler D, Schlummer M, Welle F, Strigl E. The newly developed Plasma Emission Detector with Echelle Spectrometer (EPED) in combination with GC as a tool to analyse the emission of unknown fluorinated substances from consumer products and food contact materials. In: 2nd International Workshop on New Developments of Fluorinated Surfactants; 2010; Idstein, Germany.

[69] Wolf N, Müller L, Enge S, Ungethüm T, Simat TJ. Analysis of PFAS and further VOC from fluoropolymer-coated cookware by thermal desorption-gas chromatography-mass spectrometry (TD-GC-MS). Food Addit Contam. 2024;71(12):1663–78. 10.1080/19440049.2024.2406007. 10.1080/19440049.2024.2406007 39311776

[70] Martínez-Pérez-Cejuela H, Williams ML, McLeod C, Gionfriddo E. Effective preconcentration of volatile per-and polyfluoroalkyl substances from gas and aqueous phase via solid phase microextraction. Anal Chim Acta. 2025;1345:343746. 10.1016/j.aca.2025.343746. 40015785 10.1016/j.aca.2025.343746

[71] AMCAN. Thermal desorption part 1: introduction and instrumentation. Anal Methods. 2020;12(26):3425–8. 10.1039/D0AY90082F. 32930231 10.1039/d0ay90082f

[72] Habib A, Noriega Landa E, Holbrook KL, Chacon AA, Lee W-Y. Green analytical method for perfluorocarboxylic acids (PFCAs) in water of stir bar sorptive extraction coupled with thermal desorption–gas chromatography—mass spectroscopy. Water. 2024;16(17):2543. 10.3390/w16172543.

[73] Corviseri MC, Dos Santos Polidoro A, Stevanin C, Pasti L, Franchina FA. Development and optimization of PFAS extraction in soil’s headspace followed by multidimensional gas chromatography and mass spectrometry. Anal Bioanal Chem. 2025;75:1–9. 10.1007/s00216-025-06266-4. 10.1007/s00216-025-06266-4 PMC13144168 41398091

[74] Corviseri MC, Polidoro A, De Poli M, Stevanin C, Chenet T, D’Anna C, et al. Targeted determination of volatile fluoroalkyl pollutants and non-targeted screening for environmental monitoring. Talanta. 2025;76:127944. 10.1016/j.talanta.2025.127944. 10.1016/j.talanta.2025.127944 40120512

[75] Roszkowska A, Yu M, Pawliszyn J. In vivo SPME for bioanalysis in environmental monitoring and toxicology. In: A New Paradigm for Environmental Chemistry and Toxicology: From Concepts to Insights. Springer; 2019. p. 23–31. 10.1007/978-981-13-9447-8_3.

[76] Jia S, Marques Dos Santos M, Li C, Snyder SA. Recent advances in mass spectrometry analytical techniques for per-and polyfluoroalkyl substances (PFAS). Anal Bioanal Chem. 2022;414(9):2795–807. 10.1007/s00216-022-03905-y. 35132477 10.1007/s00216-022-03905-y

[77] Alzaga R, Bayona J. Determination of perfluorocarboxylic acids in aqueous matrices by ion-pair solid-phase microextraction–in-port derivatization–gas chromatography–negative ion chemical ionization mass spectrometry. J Chromatogr A. 2004;1042(1–2):155–62. 10.1016/j.chroma.2004.05.015. 15296400 10.1016/j.chroma.2004.05.015

[78] Bach C, Boiteux V, Hemard J, Colin A, Rosin C, Munoz J-F, et al. Simultaneous determination of perfluoroalkyl iodides, perfluoroalkane sulfonamides, fluorotelomer alcohols, fluorotelomer iodides and fluorotelomer acrylates and methacrylates in water and sediments using solid-phase microextraction-gas chromatography/mass spectrometry. J Chromatogr A. 2016;1448:98–106. 10.1016/j.chroma.2016.04.025. 27125188 10.1016/j.chroma.2016.04.025

[79] Monteleone M, Naccarato A, Sindona G, Tagarelli A. A rapid and sensitive assay of perfluorocarboxylic acids in aqueous matrices by headspace solid phase microextraction–gas chromatography–triple quadrupole mass spectrometry. J Chromatogr A. 2012;1251:160–8. 10.1016/j.chroma.2012.06.033. 22762954 10.1016/j.chroma.2012.06.033

[80] Nickerson A, Maizel AC, Kulkarni PR, Adamson DT, Kornuc JJ, Higgins CP. Enhanced extraction of AFFF-associated PFASs from source zone soils. Environ Sci Technol. 2020;54(8):4952–62. 10.1021/acs.est.0c00792. 32200626 10.1021/acs.est.0c00792

[81] Joseph KM, Boatman AK, Dodds JN, Kirkwood-Donelson KI, Ryan JP, Zhang J, et al. Multidimensional library for the improved identification of per-and polyfluoroalkyl substances (PFAS). Sci Data. 2025;12(1):150. 10.1038/s41597-024-04363-0. 39863618 10.1038/s41597-024-04363-0 PMC11763048

[82] Backe WJ, Day TC, Field JA. Zwitterionic, cationic, and anionic fluorinated chemicals in aqueous film forming foam formulations and groundwater from US military bases by nonaqueous large-volume injection HPLC-MS/MS. Environ Sci Technol. 2013;47(10):5226–34. 10.1021/es3034999. 23590254 10.1021/es3034999

[83] Munoz G, Duy SV, Labadie P, Botta F, Budzinski H, Lestremau F, et al. Analysis of zwitterionic, cationic, and anionic poly-and perfluoroalkyl surfactants in sediments by liquid chromatography polarity-switching electrospray ionization coupled to high resolution mass spectrometry. Talanta. 2016;152:447–56. 10.1016/j.talanta.2016.02.021. 26992541 10.1016/j.talanta.2016.02.021

[84] Peng H, Hu K, Zhao F, Hu J. Derivatization method for sensitive determination of fluorotelomer alcohols in sediment by liquid chromatography–electrospray tandem mass spectrometry. J Chromatogr A. 2013;1288:48–53. 10.1016/j.chroma.2013.02.085. 23523067 10.1016/j.chroma.2013.02.085

[85] Portolés T, Rosales LE, Sancho JV, Santos FJ, Moyano E. Gas chromatography–tandem mass spectrometry with atmospheric pressure chemical ionization for fluorotelomer alcohols and perfluorinated sulfonamides determination. J Chromatogr A. 2015;1413:107–16. 10.1016/j.chroma.2015.08.016. 26298605 10.1016/j.chroma.2015.08.016

[86] Ayala-Cabrera JF, Javier Santos F, Moyano E. Negative-ion atmospheric pressure ionisation of semi-volatile fluorinated compounds for ultra-high-performance liquid chromatography tandem mass spectrometry analysis. Anal Bioanal Chem. 2018;410(20):4913–24. 10.1007/s00216-018-1138-z. 29796902 10.1007/s00216-018-1138-z

[87] Ayala-Cabrera J, Moyano E, Santos F. Gas chromatography and liquid chromatography coupled to mass spectrometry for the determination of fluorotelomer olefins, fluorotelomer alcohols, perfluoroalkyl sulfonamides and sulfonamido-ethanols in water. J Chromatogr A. 2020;1609:460463. 10.1016/j.chroma.2019.460463. 31447206 10.1016/j.chroma.2019.460463

[88] Lauria MZ, Shi X, Haque F, Plassmann M, Roos A, Simon M, et al. Discovery of fluorotelomer sulfones in the blubber of Greenland killer whales (*Orcinus orca*). Environ Sci Technol Lett. 2025;12(9):1218–24. 10.1021/acs.estlett.5c00516. 40951866 10.1021/acs.estlett.5c00516 PMC12424159

[89] Seró R, Ayala-Cabrera JF, Santos F, Moyano E. Paper spray-atmospheric pressure photoionization-high resolution mass spectrometry for the direct analysis of neutral fluorinated compounds in waterproof impregnation sprays. Anal Chim Acta. 2022;1204:339720. 10.1016/j.aca.2022.339720. 35397912 10.1016/j.aca.2022.339720

[90] Cai Y, Kingery D, McConnell O, Bach AC. Advantages of atmospheric pressure photoionization mass spectrometry in support of drug discovery. Rapid Commun Mass Spectrom. 2005;19(12):1717–24. 10.1002/rcm.1981. 15912481 10.1002/rcm.1981

[91] Ayala-Cabrera J, Contreras-Llin A, Moyano E, Santos F. A novel methodology for the determination of neutral perfluoroalkyl and polyfluoroalkyl substances in water by gas chromatography-atmospheric pressure photoionisation-high resolution mass spectrometry. Anal Chim Acta. 2020;1100:97–106. 10.1016/j.aca.2019.12.004. 31987157 10.1016/j.aca.2019.12.004

[92] Guo W, Zhang Y, Wang Y, Zhu L, Cai Z. A comprehensive, simple, robust, and solvent-free method covering ultrashort-to long-chain PFAS in atmospheric samples. Anal Chem. 2025;97(27):14838–46. 10.1021/acs.analchem.5c03123. 40598762 10.1021/acs.analchem.5c03123 PMC12268817

[93] Shi Q, Zhang X, Liu X, Yan C, Lu S. Visualization of PFOA accumulation and its effects on phospholipid in zebrafish liver by MALDI imaging. Anal Bioanal Chem. 2024;416(10):2493–501. 10.1007/s00216-024-05214-y. 38451276 10.1007/s00216-024-05214-y

[94] Tanim-Al Hassan M, Chen X, Fnu PIJ, Osonga FJ, Sadik OA, Li M, et al. Rapid detection of per-and polyfluoroalkyl substances (PFAS) using paper spray-based mass spectrometry. J Hazard Mater. 2024;465:96. 10.1016/j.jhazmat.2023.133366. 10.1016/j.jhazmat.2023.133366 38185081

[95] Huang Z-Y, Hu MJ, Hung L-I, Shiu R-F, Lin C-H, Lee C. Bubble preconcentration coupled with SALDI-TOF MS for rapid detection of PFAS in environmental water. Anal Bioanal Chem. 2026;418(3):783–91. 10.1007/s00216-025-06231-1. 41240085 10.1007/s00216-025-06231-1

[96] Miles L, Calder H, Warner N, Riccardino G, Ladak A, Cavagnino D, et al., editors. Analysis of PFAS in indoor air using thermal desorption coupled to gas chromatography – mass spectrometry (TD-GC-MS/MS). 10.1039/d4ea00084f

[97] Hayes H, Lutes C, Watson N, Benton D, Hanigan DJ, McCoy S, et al. Laboratory development and validation of vapor phase PFAS methods for soil gas, sewer gas, and indoor air. Environ Sci Atmos. 2025;5(1):94–109. 10.1039/D4EA00084F. 40547103 10.1039/D4EA00084F PMC12180925

[98] Goukeh MN, Abichou T, Tang Y. Measurement of fluorotelomer alcohols based on solid phase microextraction followed by gas chromatography-mass spectrometry and its application in solid waste study. Chemosphere. 2023;345:140460. 10.1016/j.chemosphere.2023.140460. 37852384 10.1016/j.chemosphere.2023.140460

[99] Parker BA, Knappe DR, Titaley IA, Wanzek TA, Field JA. Tools for understanding and predicting the affinity of per-and polyfluoroalkyl substances for anion-exchange sorbents. Environ Sci Technol. 2022;56(22):15470–7. 10.1021/acs.est.1c08345. 36265138 10.1021/acs.est.1c08345

[100] Folorunsho O, Bogush A, Kourtchev I. A new on-line SPE LC-HRMS method for simultaneous analysis of selected emerging contaminants in surface waters. Anal Methods. 2023;15(3):284–96. 10.1039/D2AY01574A. 36541663 10.1039/d2ay01574a

[101] Androulakakis A, Alygizakis N, Bizani E, Thomaidis NS. Current progress in the environmental analysis of poly-and perfluoroalkyl substances (PFAS). Environ Sci Adv. 2022;1(5):705–24. 10.1039/D2VA00147K.

[102] Sanchez-Lievanos KR, Zhang D, Simpson SM, Wijayahena MK, Rizzo G, Aguilar JMN, et al. Synthesis and evaluation of cationic porphyrin-based organic nanocages for the removal of 38 PFAS from water: experimental, theoretical, and eco-toxicological insights. ACS ES&T Engineering. 2024;5(3):701–13. 10.1021/acsestengg.4c00639.

[103] Camdzic D, Welgama HK, Crawley MR, Avasthi A, Cook TR, Aga DS. Rapid capture of per-and polyfluoroalkyl substances using a self-assembling zirconium-based metal-organic cage. ACS Appl Eng Mater. 2023;2(1):87–95. 10.1021/acsaenm.3c00592.

[104] Nahar K, Zulkarnain NA, Niven RK. A review of analytical methods and technologies for monitoring per-and polyfluoroalkyl substances (PFAS) in water. Water. 2023;15(20):3577. 10.3390/w15203577.

